# Crossover trial of reverse transcriptase inhibitors in Aicardi-Goutières syndrome

**DOI:** 10.1111/dmcn.16199

**Published:** 2024-12-04

**Authors:** Yanick J Crow, Tracy A Briggs, Despina Eleftheriou, Amitav Parida, Claire Battison, Annabel Giddings, Titouan Kennel, Richard A Parker, AGS-RTIs Study Group, Aimee Donald, Paddy Mcmaster, Jay Shetty, Katherine Jack, Alan J Quigley, Alice Burleigh, Felice D’Arco, Cheryl Hemingway, Ying Hong, Elena Moraitis, Fiona Price-Kuehne, Dr Annapurna Sudarsanam, Evangeline Wassmer, Katie Livingstone, Fraser JH Sutherland, Marie-Thérèse El-Daher, John Norrie, Hannah Ensor, Alison F Munro, Lee Murphy, Alan Maclean, Katy R Reid, Deborah Forbes, Vincent Bondet, Darragh Duffy, David PJ Hunt, Gillian I Rice

**Affiliations:** 1 Faculty of Biology, Medicine & Health, Division of Neurosciences, University of Manchester, Manchester, UK; 2 Department of Paediatrics, North Manchester General Hospital, Manchester, UK; 3 Department of Child Life and Health, The University of Edinburgh, Edinburgh, UK; 4 Department of Paediatric Neurosciences, Royal Hospital for Children and Young People, Edinburgh, UK; 5 Muir Maxwell Epilepsy Centre, Centre for Clinical Brain Sciences, The University of Edinburgh, Edinburgh, UK; 6 Radiology, Royal Hospital for Children and Young People, Edinburgh, UK; 7 Infection, Immunity and Inflammation Department, University College London Great Ormond Street Institute of Child Health, London, UK; 8 Department of Radiology, Great Ormond Street Hospital for Children, London, UK; 9 Department of Paediatric Neurology, Great Ormond Street Hospital for Children, London, UK; 10 Paediatric Rheumatology Department, Great Ormond Street Hospital for Children NHS Foundation Trust, London, UK; 11 Department of Paediatric Neurology, Birmingham Children’s Hospital, Birmingham Women and Children’s Hospital Foundation Trust, Birmingham, UK; 12 Aston Neuroscience Institute, College of Health and Life Sciences, Aston University, Birmingham, UK; 13 MRC Human Genetics Unit, Institute of Genetics and Cancer, University of Edinburgh, Edinburgh, UK; 14 Edinburgh Clinical Trials Unit, Usher Institute, University of Edinburgh, Edinburgh, UK; 15 Cancer Research UK Scotland Centre, Institute of Genetics and Cancer, University of Edinburgh, Edinburgh, UK; 16 Edinburgh Clinical Research Facility, University of Edinburgh, Edinburgh, UK; 17 UK Dementia Research Institute at University of Edinburgh, Edinburgh, UK; 18 Centre for Clinical Brain Sciences at University of Edinburgh, Edinburgh, UK; 19 Translational Immunology Unit, Institut Pasteur, Université de Paris Cité, F75015 Paris, France; 20 Division of Evolution, Infection and Genomics, School of Biological Sciences, Faculty of Biology, Medicine and Health, The University of Manchester, Manchester, UK; 1 MRC Human Genetics Unit, Institute of Genetics and Cancer, University of Edinburgh, Edinburgh, UK; 2 Laboratory of Neurogenetics and Neuroinflammation, Imagine Institute, INSERM UMR1163, Paris, France; 3 Lydia Becker Institute of Immunology and Inflammation, Faculty of Biology, Medicine and Health, Manchester Academic Health Science Centre, The University of Manchester, Manchester, UK; 4 Department of Genomic Medicine, St Marys Hospital, Manchester Foundation Trust, Manchester, UK; 5 Division of Evolution, Infection and Genomics, School of Biological Sciences, Faculty of Biology, Medicine and Health, The University of Manchester, Manchester, UK; 6 Infection, Immunity and Inflammation Department, University College London Great Ormond Street Institute of Child Health, London, UK; 7 Paediatric Rheumatology Department, Great Ormond Street Hospital for Children NHS Foundation Trust, London, UK; 8 Department of Paediatric Neurology, Birmingham Children’s Hospital, Birmingham Women and Children’s Hospital Foundation Trust, Birmingham, UK; 9 Edinburgh Clinical Trials Unit, Usher Institute, University of Edinburgh, Edinburgh, UK

## Abstract

**Aim:**

Extend the findings of a previous clinical trial suggesting combined abacavir (ABC), lamivudine (3TC) and zidovudine (AZT) reduces type I interferon (IFN) signalling in Aicardi-Goutières syndrome (AGS).

**Methods:**

Open label, non-placebo-controlled phase II clinical trial (NCT04731103) in patients <16 years with any of five AGS genotypes. Assessment of ABC or 3TC individually, or of combined ABC+3TC+AZT, on IFN stimulated gene (ISG) expression (primary outcome) and IFN-alpha protein (secondary outcome) in blood.

**Results:**

Thirteen patients were recruited. Compliance was poor in the ABC+3TC+AZT arm. No statistically significant effects were observed with ABC or 3TC, or with ABC+3TC+AZT over six weeks. A statistically significant reduction of ISG expression was recorded after three weeks of ABC+3TC+AZT, which was not mirrored by changes in IFN-alpha protein.

**Interpretation:**

There is insufficient evidence that ABC or 3TC is either effective or ineffective in reducing type I IFN signalling in AGS over six weeks. The effect of ABC+3TC+AZT at three weeks supports data from a previous clinical trial of the effect of ABC+3TC+AZT in reducing type I IFN signalling, although there was insufficient evidence of an effect at six weeks. Time to local R&D approval, and to Sponsor authorisation following R&D approval, severely limited patient recruitment.

**Funding:**

The trial was funded by the UK Medical Research Council (MRC) through an Experimental Medicine Challenge Grant award (Grant Ref: MR/S034676/1).

## Introduction

Aicardi-Goutières syndrome (AGS) is a Mendelian inborn error of immunity particularly affecting the brain and associated with significant childhood morbidity and mortality. The pathogenesis of the syndrome is hypothesized to relate to a misrepresentation of self-derived nucleic acids as non-self, and the subsequent induction of a type I interferon (IFN) mediated response simulating a chronic antiviral state.^1^ Endogenous retroelements, mobile genetic elements that can be transcribed to RNA and then to DNA by reverse transcription, constitute ~ 40% of the human genome, and have been suggested as a potential source of immunostimulatory nucleic acid in this syndrome.^2,3^

In a single center, open label pilot study involving patients with AGS, we previously administered a combination of three anti-human immunodeficiency 1 (HIV-1) nucleoside analogue reverse transcriptase inhibitors (RTIs), abacavir (ABC), lamivudine (3TC) and zidovudine (AZT) for 12 months, at doses used in HIV-1 infected children (ClinicalTrials.gov identifier NCT02363452).^4^ The primary aim was to determine the effect of treatment on the IFN score, calculated from the expression of 24 IFN stimulated genes (ISGs). IFN status was also determined by measurement of IFN-alpha protein levels in serum, plasma and cerebrospinal fluid (CSF). Eight of 11 patients recruited from a pool of 68 known patients in France with the syndrome completed the study. There was an effect of treatment on IFN signaling, with the median IFN score across all eight patients falling from 9.66 (interquartile range (IQR), 6.51 – 13.23) to 5.33 (IQR 2.76 – 10) (p<0.0001). IFN-alpha protein levels in serum and plasma, and IFN-alpha antiviral activity in CSF, were also reduced with treatment. This effect was greatest among the four patients with mutations in components of the RNase H2 complex (median score falling from 8.16 [IQR 5.41 – 11.94] to 3.5 [IQR 2.49 – 5.46]). RNA-sequencing indicated a reduction of global ISG expression after 12 months of treatment, and a return to pre-treatment levels six months after stopping therapy.

The above results support the hypothesis that certain HIV-1 RTIs can reduce IFN signaling in AGS by inhibition of reverse transcription of endogenous retroelements. To further explore this possibility, we designed a follow-up study.

## Methods

### Study design

**Figure 1:**
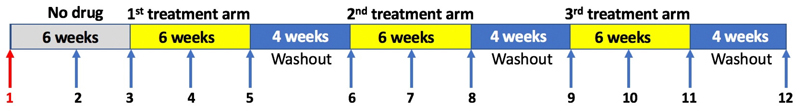
Cartoon of study timeline. Grey = no drug control period; yellow = six-week period of drug administration; blue = four-week washout period. Arrows indicate sampling points: red = screening visit; blue = within-trial sampling points. The order of allocation of the treatments ABC and 3TC was randomised between the first and second arms. All patients were scheduled to receive ABC+3TC+AZT in the third treatment arm.

This was an open label, three-arm, non-placebo-controlled phase II crossover clinical trial (ClinicalTrials.gov identifier NCT04731103), that aimed to enrol 24 children with AGS due to specified mutant genotypes. The study design consisted of a no treatment period followed by three active treatment periods each of 6 weeks duration, with each treatment period followed by a wash-out period of 4 weeks (see Figure 1). Eligible patients were randomised in a 1:1 allocation to one of two treatment sequences: one group of patients received the ABC treatment in the first period and then 3TC in the second period, while the other group of patients received 3TC first and then ABC. All patients were scheduled to receive ABC+3TC+AZT in the third treatment period. Randomisation to treatment sequences was carried out using a web-based randomisation system developed by Edinburgh Clinical Trials Unit.

### Sample size

Based on a paired t-test, a total of n=24 randomised patients provide 90% power at to detect a standardised effect size of 0.9 between treatment arms, assuming that 4 out of the 24 patients (17%) do not provide data on the primary endpoint. This calculation assumed a Bonferroni corrected two-sided 1.67% level of significance, allowing for the three multiple comparisons of active vs. no-treatment to ensure that the overall family-wise error rate is controlled at the two-sided 5% significance level. Our assumed true effect size of 0.9 is consistent with the effect sizes observed in the initial single centre study.

### Enrolment

Participants were enrolled at four centres (Edinburgh, London, Manchester and Birmingham) between September 2022 and May 2023. The study was open to residents of the United Kingdom aged between three months and less than 16 years at the time of recruitment. To be eligible for inclusion, patients had to harbour biallelic mutations in any of *TREX1*, the three components of the RNase H2 complex (*RNASEH2A, RNASEH2B, RNASEH2C*) or *SAMHD1*. Patients with mutations in *ADAR1* and *IFIH1* were not eligible for inclusion because disease in these genotypes is considered to be signalled though RNA sensing, not involving a reverse transcription step. Mutations in *LSM11* and *RNU7-1* had not been described as a cause of AGS at the time of the design of this trial. Informed consent was obtained from a parent or legal representative. Patients treated with Janus Kinase (JAK) 1 inhibitors were eligible for inclusion. No pre-screening of IFN signalling status in patients was undertaken as part of this study.

### Outcomes

IFN signalling was assessed by measuring an IFN score (primary outcome),^5^ calculated according to the expression of a panel of 24 ISGs,^6^ and IFN-alpha protein levels (secondary outcome) determined by Simoa ultrasensitive digital ELISA in patient blood (and CSF where available).^7^ Cerebral blood flow, determined by magnetic resonance imaging (MRI), was also to be considered as a secondary outcome. See Supplementary Methods for further information.

### Ethics

The study was approved by Brent Research Ethics Committee (REC) (reference: 0/LO/1150; IRAS project ID: 280253), the Medicines and Healthcare products Regulatory Agency (MHRA) (EudraCT number: 2020-003502-31), and research and development (R&D) committees local to each site (NHS Lothian; Great Ormond Street Hospital; Birmingham Women’s and Children’s NHS Foundation Trust; Manchester University Foundation Trust). The University of Edinburgh and NHS Lothian acted as joint sponsors.

### Procedures

The design of the study was informed by the results of a previous trial suggesting a significant reduction of type I IFN signalling after four weeks of therapy with ABC+3TC+AZT,^3^ thereby indicating the possibility to interrogate a drug response over this time period. In that same trial, the use of triple therapy (ABC+3TC+AZT) was based on standard treatment for children infected with HIV-1, where the risk of viral escape by mutation exists. Hypothesising that such a phenomenon would not likely apply in AGS, given the potential added value of showing an effect with the use of individual RTIs of the same class, and taking into account issues with compliance associated with combined ABC+3TC+AZT usage, the current study was designed to assess the effect on type I IFN signalling of two treatment arms involving ABC and 3TC given individually for six weeks, and a third arm of ABC+3TC+AZT which was predicted to recapitulate the effect seen previously. Each treatment arm was followed by a four-week washout period (based on the half-life of ABC^8^ and 3TC^9^). Recruitment into the study involved 12 visits (V1 - V12) over a period of 36 weeks in total. The study design also included the consented option to lumbar puncture (to assess IFN-alpha protein levels in CSF), and cerebral magnetic resonance imaging (MRI) (to assess cerebral blood flow as a proxy for a functional effect on brain function), at the start and end of one treatment arm per patient.

### Statistics

The analysis population for the primary analysis consisted of all randomised participants who received at least one dose of the active treatments in at least one of the treatment arms. All observed patient data were included in the analysis except where a patient did not take any of their allocated treatment at all during a particular treatment arm where they should have taken the treatment allocated. A treatment policy strategy (i.e. intention-to-treat) was used for patients with poor adherence to treatment, and any observations from these patients were still included in the analysis. For a complete specification of the estimand, please see the Supplementary file (Section 1).

For the primary analysis, a repeated measures normal linear mixed effect model was fitted to the primary outcome (IFN score) at all timepoints, with the following explanatory variables:

(i) Time of measurement as a continuous linear term.
(ii) Treatment received for exactly three weeks as a factor variable. No treatment was the reference category, with three dummy variables representing the three treatment arms: ABC mono, 3TC mono, and ABC+3TC+AZT combined. Note that patients were only considered to be in treatment for exactly 3 weeks at time points (visits) 4, 7, and 10.
(iii) Treatment received for exactly six weeks as a factor variable (no treatment was the reference category, with three dummy variables representing the three treatment arms: ABC mono, 3TC mono, and ABC+3TC+AZT combined). Note that patients were only considered to be in treatment for exactly 6 weeks at time points (visits) 5, 8, and 11.
(iv) Random intercept for patient.

We assumed an unstructured correlation matrix for the random effects. Results are presented as mean differences with 98.33% confidence intervals and p-values. Statistical significance was declared if p-values were below the Bonferroni-adjusted significance level of 0.0167 (two-sided), taking into account the three comparisons of each treatment with “no treatment”.

As a sensitivity analysis, we used extreme value imputation to test the sensitivity of the findings to the most extreme patterns of missing data. This analysis involved calculating the maximum and minimum observed IFN scores across all patients and timepoints. The minimum IFN score (best possible outcome) was then imputed to all missing values on no treatment, and the maximum IFN score (worst possible outcome) was imputed to all missing values on treatment. The primary analysis model was then fitted to these data.

In a secondary analysis, we investigated potential interaction effects of genotype and JAK inhibition at baseline by including interaction terms between each of these variables and treatment (parameters (ii) and (iii) above).

For the secondary outcome of IFN alpha protein levels, the same analysis method was used as for the primary analysis.

SAS version 9.4 (SAS Institute Inc., Cary, NC, USA) was used for all statistical analyses.

### Role of funding source

The funder had no role in the trial design, data collection, analysis, interpretation of results or writing of the report.

## Results

**Figure 2:**
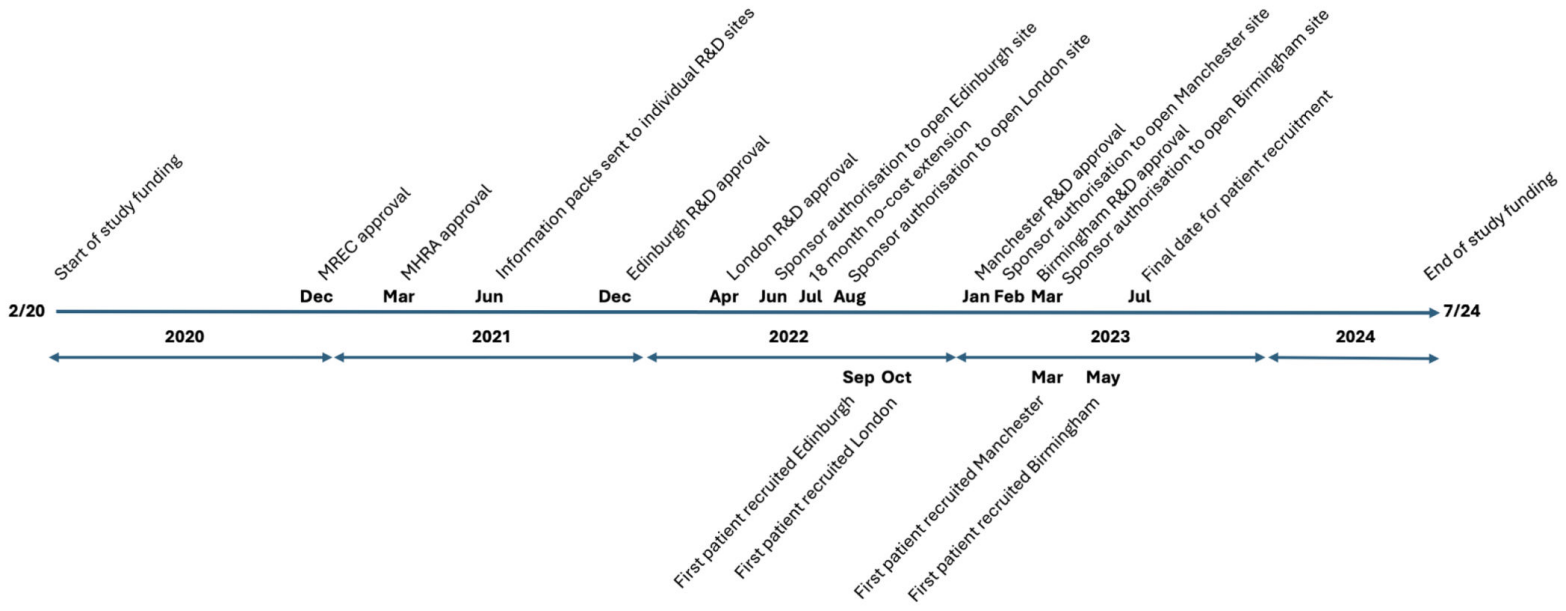
Study timeline. Regulatory milestones and date at first patient recruitment relating to the four clinical trial sites, respectively, above and below the timeline.

Study funding began in February 2020, MREC approval was obtained in December 2020, and MHRA approval in March 2021 (Figure 2). Time to R&D approval, following dispatch of information packs to all four individual sites, ranged between six to 21 months, with the time from R&D approval to sponsor authorisation for site opening a further one to six months. Time to first patient screening following sponsor authorisation to open was less than three months at all sites.

**Figure 3:**
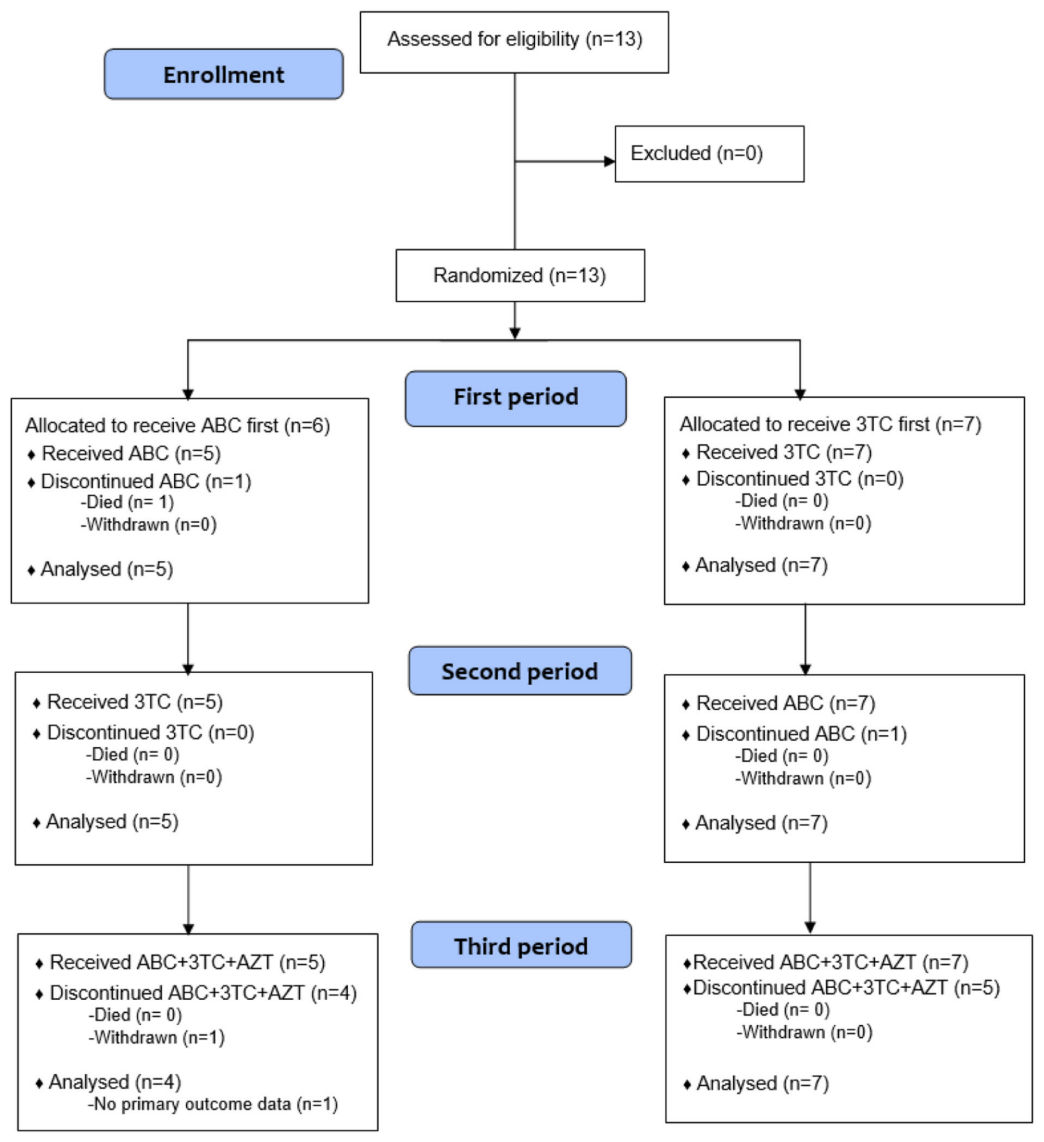
CONSORT Flow diagram

The CONSORT Flow diagram is shown in Figure 3. Thirteen patients were recruited, five mutated in *RNASEH2B*, three in *TREX1*, three in *SAMHD1*, and two in *RNASEH2C* (Table 1; Supplementary table S1). There were nine males and four females, with an age range of 1 to 15 years. Three patients were taking JAK inhibition throughout the study period. Two patients, both mutated in *RNASEH2B*, and both taking a JAK inhibitor, did not demonstrate abnormal IFN scores at baseline.

Ten serious adverse events were recorded, of which one did not occur during a treatment period, and of the remainder, none were considered to be directly related to treatment. One patient died during the second treatment arm of the study, and one patient was withdrawn at the beginning of the third treatment arm due to an intestinal perforation.

Compliance was poor in the final treatment arm (Table 1; Supplementary figure S1), with only four of 12 patients entering the final treatment arm able to fully tolerate the prescribed dosing for six weeks. While possible adverse effects were noted, the major issues determining compliance related to the volume of syrup per dosing and the associated bitter taste, compounded by poor oromotor coordination secondary to underlying neurological disease. These issues were particularly marked with the triple therapy where, as an example, a child weighing 15kg would be required to take a total volume of 27ml twice daily (lamivudine, 7.5ml; abacavir, 6ml; zidovudine 13.5ml).

A total of 141 IFN scores, and the same number of IFN-alpha protein levels in plasma, were recorded (Supplementary tables S2 and S3). There was a good correlation between the two measures (Supplementary figure S2). Table 2 shows the mean values for IFN score and IFN-alpha protein levels in each treatment arm. No statistically significant effects were observed with the use of either ABC or 3TC individually (Table 3). There was also no statistically significant effect of triple therapy (ABC+3TC+AZT) at six weeks (mean difference -1.90, 98.33% CI -4.43 to 0.64, p=0.072). A statistically significant reduction of the IFN score was recorded after three weeks of triple therapy (ABC+3TC+AZT) (mean difference -2.45, 98.33% CI -4.84 to -0.07, p=0.014), but this was not mirrored by changes in IFN-alpha protein levels (Table 4). The IFN score model results (triple therapy) were similar after conducting the extreme value imputation analysis (mean difference -2.57, 98.33% CI -5.78 to 0.63, p=0.054), albeit the p-value is non-significant (Supplementary table S4). Patients with the *RNASEH2B* genotype had a significantly higher IFN score after taking ABC mono for 6 weeks (mean difference 7.27, 95% CI 2.29 to 12.24, p=0.005), although this result should be interpreted with caution due to the high number of genotype interactions considered (18). Otherwise, no differences were noted between genotypes. We also observed no significant difference in treatment effects according to JAK inhibition at baseline.

Cerebral MRI and lumbar puncture were undertaken in only two patients, so that cerebral blood flow and CSF IFN-alpha protein levels were not subject to formal statistical modelling and analysis.

## Discussion

AGS is a devasting disease of childhood associated with significant morbidity and mortality. Indeed, the health burden associated with the disorder is reflected in the death of one patient during the course of this study, the poor compliance with study medication at least partially related to difficulties with swallowing, and reduced sampling in some patients due to limb contractures limiting venous access. Treatments limiting brain damage in AGS are urgently required, with the likely prerequisite of good central nervous system drug penetration. Based on a hypothesised role in inhibiting a reverse transcription step in the generation of endogenous retroelements, and given an excellent understanding of their pharmacokinetics, pharmacodynamics and safety profile across all ages, the use of RTIs in AGS is appealing (with two other clinical trials using this class of drugs in AGS currently registered at ClinicalTrials.gov: NCT05613868; NCT03304717). Notably, a previous study indicated that triple therapy (ABC+3TC+AZT), a standard regimen employed in the treatment of HIV-1, could reduce type I IFN signalling in patients with selected AGS-related genotypes. The present study was designed in light of these encouraging results, with the aim to further explore the hypothesis that endogenous retroelements represent a source of self-derived nucleic acids driving the enhanced type I IFN seen in AGS and considered central to its pathogenesis.

While patients were treated for 12 months, a notable feature of our original study was the observation of a reduction of type I IFN signalling after four weeks of therapy, indicating a possible opportunity to interrogate a drug response over this time period. Further, having seen an effect with triple therapy, we were interested to explore the potential added value of showing an effect with the use of individual RTIs of the same class. In these ways, we hoped to maximise the amount of information that could be extracted from the small number of patients available for study, while minimising the length of time – and burden for families - associated with participation in a clinical trial.

The crossover study design enabled participants to act as their own control and have the opportunity to take all three of the treatment regimes. Repeated measurements per patient maximised the amount of information available for analysis, which were fully utilised in the statistical analysis method. Thus, comparisons between each active treatment and “no treatment” were mainly within-patients rather than between-patients. Nevertheless, we acknowledge that the interpretation of the results derived in the present study is limited by the low number of recruited patients, and by the difficulties experienced with drug compliance (especially in the ABC+3TC+AZT arm).

There was insufficient evidence of the effectiveness of single therapy with ABC or 3TC in reducing type I IFN signalling over a six-week period in selected AGS genotypes, while not precluding the possibility that treatment over a longer period might be associated with an effect. In contrast to these non-significant results, a statistically significant reduction of the IFN score was recorded after three weeks of triple therapy (where compliance was less of an issue than in the final three weeks of this drug arm). Even if this effect was not mirrored by a statistically significant reduction in IFN-alpha protein levels, such a result is in keeping with the findings of our previous study.

The extreme value imputation method allowed us to test the sensitivity of the findings to the missing data, and this method has the advantage of covering all possible patterns of missing data (not just what we consider to be the most likely values). However, if results differ between the best-case and worst-case scenarios, this can make interpretation of the results challenging. Although the p-value for the effect of triple therapy became non-significant (p=0.054) after applying this method, the mean difference and confidence intervals were similar to our primary analysis, suggesting that our results were fairly robust to our assumptions regarding the missing data.

The issue with compliance encountered in this trial, particularly relating to combined therapy comprising ABC+3TC+AZT, is notable. Similar difficulties were experienced in our earlier trial, where three of 11 patients were unable to tolerate triple therapy over a 12-month period. Indeed, this latter observation was one of the reasons prompting us to consider the use of single RTIs in a follow-up study. More generally, the experience gained in the two studies suggests that the use of triple therapy in AGS patients is challenging over the longer term.

Two patients, both mutated in *RNASEH2B* (both of whom were also being treated with the JAK1/2 inhibitor baricitinib), did not demonstrate an upregulation of type I IFN signalling at baseline, an observation in keeping with earlier studies.^4^ In our previous trial, such patients were excluded by pre-screening, where a positive IFN score on at least three occasions in the six months prior to recruitment was stipulated as an inclusion criterion. For pragmatic reasons related to clinical trial regulations, a similar pre-screening strategy was not feasible in this study. Of note, post-hoc assessment of the primary outcome measure excluding these patients did not alter the results obtained (Supplementary table S5).

Even while noting the obvious impact of the COVID-19 pandemic on non-COVID-19 clinical trials during 2020, the present study highlights structural issues, also noted by others^10,11^ and particularly affecting paediatric studies^12^, that severely affected trial prosecution. Thus, taking the dispatch of information packs to local sites in June 2021, the time to R&D approval at the four sites varied between six and 21 months, with sponsor authorisation taking a further one to six months thereafter. These non-clinical delays contrast with the time to first patient screening following sponsor authorisation to open, which was less than three months at all sites. Compounding the effect on patient enrolment at certain sites due to limited staffing capacity, authorisation delays meant that recruitment at two sites was limited to a period of less than six months (i.e. 11% of an overall trial length of 54 months). Other non-patient related problems encountered included the non-availability of research MRI-time for this study at one site, and a non-negotiable decision of one Associate Medical Director to deny the option to participants of MRI and LP under sedation at another site. This latter decision resulted in a further six-month delay to site-opening while permission was sought from the REC and MHRA for an amendment of the protocol that they had sanctioned in the prior six months. While the effect of these issues on the outcome of the present trial is not possible to determine, unless resolved, such structural difficulties clearly have implications for future rare disease experimental medicine approaches in the UK^13^.

## Supplementary Material

Supplementary

## Figures and Tables

**Table 1:** General characteristics of the study cohort in each randomised group and overall

		ABC -> 3TC (N=6)	3TC -> ABC (N=7)	All (N=13)
**Sex**				
	Male	3 (50%)	6 (86%)	9 (69%)
	Female	3 (50%)	1 (14%)	4 (31%)
**Ethnicity**				
	White	1 (17%)	7 (100%)	8 (62%)
	Mixed or Multiple Ethnic Groups	1 (17%)	0	1 (8%)
	Asian	3 (50%)	0	3 (23%)
	Other ethnic group	1 (17%)	0	1 (8%)
**Genetic status**	TREX1			
		1 (17%)	2 (29%)	3 (23%)
	RNASEH2B	3 (50%)	2 (29%)	5 (38%)
	RNASEH2C	2 (33%)	0	2 (15%)
	SAMHD1	0	3 (43%)	3 (23%)
**JAK Inhibitors**	Yes			
		1 (17%)	2 (29%)	3 (23%)
	No	5 (83%)	5 (71%)	10 (77%)
**Age (year)**				
	Mean (SD)			
		8.7 (5.3)	4.7 (2.9)	6.5 (4.5)
	Median [Q1-Q3]	9 [5-13]	4 [2-8]	5 [4-9]
	Min Max	1,15	1,9	1,15
	N	6	7	13

Numbers are n(%), mean (SD) or median [Q1-Q3]

**Table 2:** Mean value on primary outcome measure (interferon (IFN) score) and secondary outcome measure (interferon (IFN) alpha protein) according to treatment arm

Timepoint	Mean – interferon (IFN) score (SD) (N=)	Mean – interferon (IFN) alpha protein (SD) (N=)
ABC at 3 weeks	6.6 (4.9) N=12	3935 (9837)N=12
ABC at 6 weeks	6.9 (3.8) N=11	1106 (1069) N=12
3TC at 3 weeks	8.0 (5.5) N=12	2052 (2108) N=12
3TC at 6 weeks	4.9 (4.1) N=11	1629 (2148) N=11
ABC+3TC+AZT at 3 weeks	4.2 (4.1) N=11	1042 (1978) N=10
ABC+3TC+AZT at 6 weeks	4.2 (4.1) N=10	10906 (31702) N=10
No treatment (average within each patient)	6.4 (4.8) N=13	2034 (1649) N=13

**Table 3:** Modelled assessment on primary outcome measure (interferon (IFN) score) according to treatment arm

Comparison (vs no treatment)	Mean difference (active vs no treatment)	98.33% CI Lower	98.33% CI Upper	p-value
ABC at 3 weeks	0.23	-1.90	2.37	0.79
ABC at 6 weeks	-0.25	-2.49	1.98	0.79
3TC at 3 weeks	1.73	-0.40	3.86	0.05
3TC at 6 weeks	-1.37	-3.59	0.85	0.14
ABC+3TC+AZT at 3 weeks	-2.45	-4.84	-0.07	0.014
ABC+3TC+AZT at 6 weeks	-1.90	-4.43	0.64	0.072

ABC = abacavir; 3TC = lamivudine; AZT = zidovudine

**Table 4:** Modelled assessment on secondary outcome measure (interferon (IFN) alpha protein) according to treatment arm

Comparison (vs no treatment)	Mean difference (active vs no treatment)	98.33% CI Lower	98.33% CI Upper	p-value
ABC at 3 weeks	1817	-3621	7255	0.51
ABC at 6 weeks	-1122	-6601	4358	0.69
3TC at 3 weeks	46	-5377	5469	0.99
3TC at 6 weeks	-568	-6230	5095	0.84
ABC+3TC+AZT at 3 weeks	-1580	-7906	4747	0.62
ABC+3TC+AZT at 6 weeks	8217	1724	14709	0.01

ABC = abacavir; 3TC = lamivudine; AZT = zidovudine

## Data Availability

Due to issues of patient confidentiality, no data are available beyond those in the published manuscript.

## What this paper adds

- Abacavir or lamivudine did not significantly reduce interferon signalling in AGS
- Triple therapy did not reduce interferon signalling in AGS at 6 weeks
- Triple therapy significantly reduced interferon signalling in AGS at 3 weeks
- Non-clinical factors negatively impacted patient recruitment to a major degree
- Structural failings represent a serious impediment to UK paediatric experimental medicine
