# Reverse transcriptase inhibitors in Aicardi–Goutières syndrome: A crossover clinical trial

**DOI:** 10.1111/dmcn.16199

**Published:** 2024-12-04

**Authors:** Yanick J. Crow, Tracy A. Briggs, Despina Eleftheriou, Amitav Parida, Claire Battison, Annabel Giddings, Titouan Kennel, Richard A. Parker, AGS‐RTIs Study Group, Aimee Donald, Paddy Mcmaster, Jay Shetty, Katherine Jack, Alan J Quigley, Alice Burleigh, Felice D’Arco, Cheryl Hemingway, Ying Hong, Elena Moraitis, Fiona Price‐Kuehne, Annapurna Sudarsanam, Evangeline Wassmer, Katie Livingstone, Fraser JH Sutherland, Marie‐Thérèse El‐Daher, John Norrie, Hannah Ensor, Alison F Munro, Lee Murphy, Alan Maclean, Katy R Reid, Deborah Forbes, Vincent Bondet, Darragh Duffy, David PJ Hunt, Gillian I Rice

**Affiliations:** ^1^ MRC Human Genetics Unit, Institute of Genetics and Cancer University of Edinburgh Edinburgh UK; ^2^ Laboratory of Neurogenetics and Neuroinflammation Imagine Institute, INSERM UMR1163 Paris France; ^3^ Lydia Becker Institute of Immunology and Inflammation, Faculty of Biology, Medicine and Health, Manchester Academic Health Science Centre The University of Manchester Manchester UK; ^4^ Department of Genomic Medicine St Marys Hospital, Manchester Foundation Trust Manchester UK; ^5^ Division of Evolution, Infection and Genomics, School of Biological Sciences, Faculty of Biology, Medicine and Health The University of Manchester Manchester UK; ^6^ Infection, Immunity and Inflammation Department University College London Great Ormond Street Institute of Child Health London UK; ^7^ Paediatric Rheumatology Department Great Ormond Street Hospital for Children NHS Foundation Trust London UK; ^8^ Department of Paediatric Neurology Birmingham Children's Hospital, Birmingham Women and Children's Hospital Foundation Trust Birmingham UK; ^9^ Edinburgh Clinical Trials Unit, Usher Institute University of Edinburgh Edinburgh UK

## Abstract

**Aim:**

To extend the findings of a previous clinical trial suggesting combined abacavir (ABC), lamivudine (3TC), and zidovudine (AZT) reduces type I interferon (IFN) signalling in Aicardi–Goutières syndrome (AGS).

**Method:**

This was an open label, non‐placebo‐controlled phase II clinical trial (NCT04731103) in patients less than 16 years with any of five AGS genotypes. The effect of ABC or 3TC individually, or of combined ABC + 3TC + AZT, on IFN‐stimulated gene (ISG) expression (primary outcome) and IFN‐alpha protein (secondary outcome) in blood was assessed.

**Results:**

Thirteen patients were recruited. Compliance was poor in the ABC + 3TC + AZT arm. No statistically significant effects were observed with ABC or 3TC, or with ABC + 3TC + AZT over 6 weeks. A statistically significant reduction of ISG expression was recorded after 3 weeks of ABC + 3TC + AZT, which was not mirrored by changes in IFN‐alpha protein.

**Interpretation:**

There is insufficient evidence that ABC or 3TC is either effective or ineffective in reducing type I IFN signalling in AGS over 6 weeks. The effect of ABC + 3TC + AZT at 3 weeks supports data from a previous clinical trial of the effect of ABC + 3TC + AZT in reducing type I IFN signalling, although there was insufficient evidence of an effect at 6 weeks. Time to local research and development (R&D) approval, and to sponsor authorization after R&D approval, severely limited patient recruitment.

## Graphical abstract

Viral nucleic acid recognition induces a physiological type I interferon mediated innate immune response. In some cases of Aicardi‐Goutières syndrome, this same response is proposed to be triggered by self‐derived nucleic acid generated through reverse transcription. Here, reverse transcriptase inhibitors were assessed in such cases.

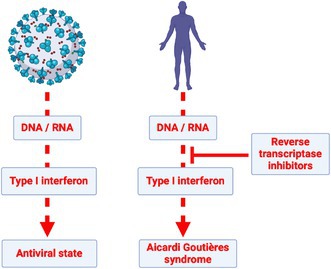

Plain language summary: https://onlinelibrary.wiley.com/doi/10.1111/dmcn.16222

**This original article is commented by Dale on pages** 696–697 **of this issue.**

**Abbreviations**
3TC: lamivudine
ABC: abacavir
AGS: Aicardi–Goutières syndrome
AZT: zidovudine
CSF: cerebrospinal fluid
HIV‐1: human immunodeficiency 1
IFN: interferon
ISG: IFN‐stimulated gene
JAK: Janus kinase
MHRA: Medicines and Healthcare products Regulatory Agency
R&D: research and development
RNA: ribonucleic acid
RTI: reverse transcriptase inhibitor

**What this paper adds**

- Abacavir or lamivudine did not significantly reduce interferon signalling in Aicardi–Goutières syndrome (AGS).
- Triple therapy did not reduce interferon signalling in AGS at 6 weeks.
- Triple therapy significantly reduced interferon signalling in AGS at 3 weeks.
- Non‐clinical factors negatively impacted patient recruitment to a major degree.
- Structural failings represent a serious impediment to UK paediatric experimental medicine.

Aicardi–Goutières syndrome (AGS) is a Mendelian inborn error of immunity particularly affecting the brain and is associated with significant childhood morbidity and mortality. The pathogenesis of the syndrome is hypothesized to relate to a misrepresentation of self‐derived nucleic acids as non‐self, and the subsequent induction of a type I interferon (IFN) mediated response simulating a chronic antiviral state.^1^ Endogenous retroelements, mobile genetic elements that can be transcribed to ribonucleic acid (RNA) and then to DNA by reverse transcription, constitute around 40% of the human genome, and have been suggested as a potential source of immunostimulatory nucleic acid in this syndrome.^2^, ^3^

In a single centre, open label pilot study involving patients with AGS, we previously administered a combination of three anti‐human immunodeficiency 1 (HIV‐1) nucleoside analogue reverse transcriptase inhibitors (RTIs), abacavir (ABC), lamivudine (3TC), and zidovudine (AZT) for 12 months, at doses used in HIV‐1 infected children (ClinicalTrials.gov identifier NCT02363452).^4^ The primary aim was to determine the effect of treatment on the IFN score, calculated from the expression of 24 IFN‐stimulated genes (ISGs). IFN status was also determined by measurement of IFN‐alpha protein levels in serum, plasma, and cerebrospinal fluid (CSF). Eight of 11 patients recruited from a pool of 68 known patients in France with the syndrome completed the study. There was an effect of treatment on IFN signalling, with the median IFN score across all eight patients falling from 9.66 (interquartile range [IQR] 6.51–13.23) to 5.33 (IQR 2.76–10) (*p* < 0.0001). IFN‐alpha protein levels in serum and plasma, and IFN‐alpha antiviral activity in CSF, were also reduced with treatment. This effect was greatest among the four patients with mutations in components of the RNase H2 complex (median score falling from 8.16 [IQR 5.41–11.94] to 3.5 [IQR 2.49–5.46]). RNA‐sequencing indicated a reduction of global ISG expression after 12 months of treatment, and a return to pretreatment levels 6 months after stopping therapy.

The above results support the hypothesis that certain HIV‐1 RTIs can reduce IFN signalling in AGS by inhibition of reverse transcription of endogenous retroelements. To further explore this possibility, we designed a follow‐up study.

## METHOD

### Study design

This was an open label, three‐arm, non‐placebo‐controlled phase II crossover clinical trial (ClinicalTrials.gov identifier NCT04731103), that aimed to enrol 24 children with AGS due to specified mutant genotypes. The study design consisted of a no treatment period followed by three active treatment periods each of 6 weeks duration, with each treatment period followed by a washout period of 4 weeks (see Figure S1). Eligible patients were randomized in a 1:1 allocation to one of two treatment sequences: one group of patients received the ABC treatment in the first period and then 3TC in the second period, while the other group of patients received 3TC first and then ABC. All patients were scheduled to receive ABC + 3TC + AZT in the third treatment period. Randomization to treatment sequences was carried out using a web‐based randomization system developed by Edinburgh Clinical Trials Unit.

### Sample size

Based on a paired *t*‐test, a total of 24 randomized patients provided 90% power to detect a standardized effect size of 0.9 between treatment arms, assuming that four out of the 24 patients do not provide data on the primary endpoint. This calculation assumed a Bonferroni corrected two‐sided 1.67% level of significance, allowing for the three multiple comparisons of active versus no treatment to ensure that the overall family‐wise error rate is controlled at the two‐sided 5% significance level. Our assumed true effect size of 0.9 is consistent with the effect sizes observed in the initial single centre study.

### Enrolment

Participants were enrolled at four centres (Edinburgh, London, Manchester, and Birmingham) between September 2022 and May 2023. The study was open to residents of the UK aged between 3 months and less than 16 years at the time of recruitment. To be eligible for inclusion, patients had to harbour biallelic mutations in any of *TREX1*, the three components of the RNase H2 complex (*RNASEH2A*, *RNASEH2B*, *RNASEH2C*) or *SAMHD1*. Patients with mutations in *ADAR1* and *IFIH1* were not eligible for inclusion because disease in these genotypes is considered to be signalled though RNA sensing, not involving a reverse transcription step. Mutations in *LSM11* and *RNU7‐1* had not been described as a cause of AGS at the time of the design of this trial. Written informed consent was obtained from a parent or legal representative. Patients treated with Janus kinase (JAK) 1 inhibitors were eligible for inclusion. No prescreening of IFN signalling status in patients was undertaken as part of this study.

### Outcomes

IFN signalling was assessed by measuring an IFN score (primary outcome),^5^ calculated according to the expression of a panel of 24 ISGs,^6^ and IFN‐alpha protein levels (secondary outcome) determined by Simoa ultrasensitive digital enzyme‐linked immunosorbent assay in patient blood (and CSF where available).^7^ Cerebral blood flow, determined by magnetic resonance imaging (MRI), was also to be considered as a secondary outcome (see Appendix S1 for further information).

### Ethics

The study was approved by Brent Research Ethics Committee (reference: 0/LO/1150; IRAS project ID: 280253), the Medicines and Healthcare products Regulatory Agency (MHRA) (EudraCT number: 2020–003502‐31), and research and development (R&D) committees local to each site (NHS Lothian; Great Ormond Street Hospital; Birmingham Women's and Children's NHS Foundation Trust; Manchester University Foundation Trust). The University of Edinburgh and NHS Lothian acted as joint sponsors.

### Procedures

The design of the study was informed by the results of a previous trial suggesting a significant reduction of type I IFN signalling after 4 weeks of therapy with ABC + 3TC + AZT,^3^ thereby indicating the possibility to interrogate a drug response over this time period. In that same trial, the use of triple therapy (ABC + 3TC + AZT) was based on standard treatment for children infected with HIV‐1, where the risk of viral escape by mutation exists. Hypothesizing that such a phenomenon would not likely apply in AGS, given the potential added value of showing an effect with the use of individual RTIs of the same class, and taking into account issues with compliance associated with combined ABC + 3TC + AZT usage, the current study was designed to assess the effect on type I IFN signalling of two treatment arms involving ABC and 3TC given individually for 6 weeks, and a third arm of ABC + 3TC + AZT which was predicted to recapitulate the effect seen previously. Each treatment arm was followed by a 4‐week washout period (based on the half‐life of ABC^8^ and 3TC^9^). Recruitment into the study involved 12 visits (V1–V12) over a period of 36 weeks in total. The study design also included the consented option to lumbar puncture (to assess IFN‐alpha protein levels in CSF), and cerebral MRI (to assess cerebral blood flow as a proxy for a functional effect on brain function), at the start and end of one treatment arm per patient.

### Statistics

The analysis population for the primary analysis consisted of all randomized participants who received at least one dose of the active treatments in at least one of the treatment arms. All observed patient data were included in the analysis except where a patient did not take any of their allocated treatment at all during a particular treatment arm where they should have taken the treatment allocated. A treatment policy strategy (i.e. intention‐to‐treat) was used for patients with poor adherence to treatment, and any observations from these patients were still included in the analysis. For a complete specification of the estimand, please see Appendix S2.

For the primary analysis, a repeated measures normal linear mixed effects model was fitted to the primary outcome (IFN score) at all timepoints, with the following explanatory variables: (1) Time of measurement as a continuous linear term. (2) Treatment received for exactly 3 weeks as a factor variable. No treatment was the reference category, with three dummy variables representing the three treatment arms: ABC mono, 3TC mono, and ABC + 3TC + AZT combined. Note that patients were only considered to be in treatment for exactly 3 weeks at time points (visits) 4, 7, and 10. (3) Treatment received for exactly 6 weeks as a factor variable (no treatment was the reference category, with three dummy variables representing the three treatment arms: ABC mono, 3TC mono, and ABC + 3TC + AZT combined). Note that patients were only considered to be in treatment for exactly 6 weeks at time points (visits) 5, 8, and 11. (4) Random intercept for patient.

We assumed an unstructured correlation matrix for the random effects. Results are presented as mean differences with 98.33% confidence intervals and *p*‐values. Statistical significance was declared if *p*‐values were below the Bonferroni‐adjusted significance level of 0.0167 (two‐sided), taking into account the three comparisons of each treatment with ‘no treatment’.

As a sensitivity analysis, we used extreme value imputation to test the sensitivity of the findings to the most extreme patterns of missing data. This analysis involved calculating the maximum and minimum observed IFN scores across all patients and timepoints. The minimum IFN score (best possible outcome) was then imputed to all missing values on no treatment, and the maximum IFN score (worst possible outcome) was imputed to all missing values on treatment. The primary analysis model was then fitted to these data.

In a secondary analysis, we investigated potential interaction effects of genotype and JAK inhibition at baseline by including interaction terms between each of these variables and treatment (parameters 2 and 3 above).

For the secondary outcome of IFN‐alpha protein levels, the same analysis method was used as for the primary analysis.

SAS version 9.4 (SAS Institute, Cary, NC, USA) was used for all statistical analyses.

## RESULTS

Study funding began in February 2020, medical research ethics committee approval was obtained in December 2020, and MHRA approval in March 2021 (Figure S2). Time to R&D approval, after dispatch of information packs to all four individual sites, ranged between 6 to 21 months, with the time from R&D approval to sponsor authorization for site opening a further 1 to 6 months. Time to first patient screening after sponsor authorization to open was less than 3 months at all sites.

The CONSORT flow diagram is shown in Figure S3. Thirteen patients were recruited, five mutated in *RNASEH2B*, three in *TREX1*, three in *SAMHD1*, and two in *RNASEH2C* (Table 1; Table S1). There were nine males and four females, with an age range of 1 year to 15 years. Three patients were taking JAK inhibition throughout the study period. Two patients, both mutated in *RNASEH2B*, and both taking a JAK inhibitor, did not demonstrate abnormal IFN scores at baseline.

**TABLE 1:** General characteristics of the study cohort in each randomized group and overall.

	Changing from ABC to 3TC (*n* = 6)	Changing from 3TC to ABC (*n* = 7)	All (*n* = 13)
**Sex**			
Male	3 (50%)	6 (86%)	9 (69%)
Female	3 (50%)	1 (14%)	4 (31%)
**Ethnicity**			
White	1 (17%)	7 (100%)	8 (62%)
Mixed or multiple ethnic groups	1 (17%)	0	1 (8%)
Asian	3 (50%)	0	3 (23%)
Other ethnic group	1 (17%)	0	1 (8%)
**Genetic status**			
TREX1	1 (17%)	2 (29%)	3 (23%)
RNASEH2B	3 (50%)	2 (29%)	5 (38%)
RNASEH2C	2 (33%)	0	2 (15%)
SAMHD1	0	3 (43%)	3 (23%)
**JAK inhibitors**			
Yes	1 (17%)	2 (29%)	3 (23%)
No	5 (83%)	5 (71%)	10 (77%)
**Age (years)**			
Mean (SD)	8.7 (5.3)	4.7 (2.9)	6.5 (4.5)
Median (Q1–Q3)	9 (5–13)	4 (2–8)	5 (4–9)
Min, max	1, 15	1, 9	1, 15
*n*	6	7	13

*Note*: Numbers are *n* (%), mean (SD), or median (Q1–Q3).

Ten serious adverse events were recorded, of which one did not occur during a treatment period, and of the remainder, none were considered to be directly related to treatment. One patient died during the second treatment arm of the study, and one patient was withdrawn at the beginning of the third treatment arm because of an intestinal perforation.

Compliance was poor in the final treatment arm (Table 1; Figure S4), with only four of 12 patients entering the final treatment arm able to fully tolerate the prescribed dosing for 6 weeks. While possible adverse effects were noted, the major issues determining compliance related to the volume of syrup per dosing and the associated bitter taste, compounded by poor oromotor coordination secondary to underlying neurological disease. These issues were particularly marked with the triple therapy where, as an example, a child weighing 15 kg would be required to take a total volume of 27 mL twice daily (lamivudine, 7.5 mL; abacavir, 6 mL; zidovudine 13.5 mL).

A total of 141 IFN scores, and the same number of IFN‐alpha protein levels in plasma, were recorded (Tables S2 and S3). There was a good correlation between the two measures (Figure S5). Table 2 shows the mean values for IFN score and IFN‐alpha protein levels in each treatment arm. No statistically significant effects were observed with the use of either ABC or 3TC individually (Table 3). There was also no statistically significant effect of triple therapy (ABC + 3TC + AZT) at 6 weeks (mean difference − 1.90, 98.33% confidence interval [CI] –4.43 to 0.64, *p* = 0.072). A statistically significant reduction of the IFN score was recorded after 3 weeks of triple therapy (ABC + 3TC + AZT) (mean difference − 2.45, 98.33% CI –4.84 to −0.07, *p* = 0.014), but this was not mirrored by changes in IFN‐alpha protein levels (Table 4). The IFN score model results (triple therapy) were similar after conducting the extreme value imputation analysis (mean difference − 2.57, 98.33% CI –5.78 to 0.63, *p* = 0.054), albeit the *p*‐value is non‐significant (Table S4). Patients with the *RNASEH2B* genotype had a significantly higher IFN score after taking ABC mono for 6 weeks (mean difference 7.27, 95% CI 2.29 to 12.24, *p* = 0.005), although this result should be interpreted with caution because of the high number of genotype interactions considered (*n* = 18). Otherwise, no differences were noted between genotypes. We also observed no significant difference in treatment effects according to JAK inhibition at baseline.

**TABLE 2:** Mean value on primary outcome measure (interferon score) and secondary outcome measure (interferon alpha protein) according to treatment arm.

Timepoint	Mean–interferon score (SD)	Mean–interferon alpha protein (SD)
ABC at 3 weeks	6.6 (4.9) *n* = 12	3935 (9837) *n* = 12
ABC at 6 weeks	6.9 (3.8) *n* = 11	1106 (1069) *n* = 12
3TC at 3 weeks	8.0 (5.5) *n* = 12	2052 (2108) *n* = 12
3TC at 6 weeks	4.9 (4.1) *n* = 11	1629 (2148) *n* = 11
ABC + 3TC + AZT at 3 weeks	4.2 (4.1) *n* = 11	1042 (1978) *n* = 10
ABC + 3TC + AZT at 6 weeks	4.2 (4.1) *n* = 10	10 906 (31702) *n* = 10
No treatment (average within each patient)	6.4 (4.8) *n* = 13	2034 (1649) *n* = 13

Abbreviations: 3TC, lamivudine; ABC, abacavir; AZT, zidovudine.

**TABLE 3:** Modelled assessment on primary outcome measure (interferon score) according to treatment arm.

Comparison (vs. no treatment)	Mean difference (active vs. no treatment)	98.33% CI lower	98.33% CI upper	*p*
ABC at 3 weeks	0.23	−1.90	2.37	0.79
ABC at 6 weeks	−0.25	−2.49	1.98	0.79
3TC at 3 weeks	1.73	−0.40	3.86	0.05
3TC at 6 weeks	−1.37	−3.59	0.85	0.14
ABC + 3TC + AZT at 3 weeks	−2.45	−4.84	−0.07	0.014
ABC + 3TC + AZT at 6 weeks	−1.90	−4.43	0.64	0.072

Abbreviations: 3TC, lamivudine; ABC, abacavir; AZT, zidovudine; CI, confidence interval.

**TABLE 4:** Modelled assessment on secondary outcome measure (interferon alpha protein) according to treatment arm.

Comparison (vs. no treatment)	Mean difference (active vs. no treatment)	98.33% CI lower	98.33% CI upper	*p*
ABC at 3 weeks	1817	−3621	7255	0.51
ABC at 6 weeks	−1122	−6601	4358	0.69
3TC at 3 weeks	46	−5377	5469	0.99
3TC at 6 weeks	−568	−6230	5095	0.84
ABC + 3TC + AZT at 3 weeks	−1580	−7906	4747	0.62
ABC + 3TC + AZT at 6 weeks	8217	1724	14709	0.01

Abbreviations: 3TC, lamivudine; ABC, abacavir; AZT, zidovudine; CI, confidence interval.

Cerebral MRI and lumbar puncture were undertaken in only two patients, so that cerebral blood flow and CSF IFN‐alpha protein levels were not subject to formal statistical modelling and analysis.

## DISCUSSION

AGS is a devasting disease of childhood associated with significant morbidity and mortality. Indeed, the health burden associated with the disorder is reflected in the death of one patient during the course of this study, the poor compliance with study medication at least partially related to difficulties with swallowing, and reduced sampling in some patients due to limb contractures limiting venous access. Treatments limiting brain damage in AGS are urgently required, with the likely prerequisite of good central nervous system drug penetration. Based on a hypothesized role in inhibiting a reverse transcription step in the generation of endogenous retroelements, and given an excellent understanding of their pharmacokinetics, pharmacodynamics, and safety profile across all ages, the use of RTIs in AGS is appealing (with two other clinical trials using this class of drugs in AGS currently registered at ClinicalTrials.gov: NCT05613868; NCT03304717). Notably, a previous study indicated that triple therapy (ABC + 3TC + AZT), a standard regimen employed in the treatment of HIV‐1, could reduce type I IFN signalling in patients with selected AGS‐related genotypes. The present study was designed in light of these encouraging results, with the aim to further explore the hypothesis that endogenous retroelements represent a source of self‐derived nucleic acids driving the enhanced type I IFN seen in AGS and considered central to its pathogenesis.

While patients were treated for 12 months, a notable feature of our original study was the observation of a reduction of type I IFN signalling after 4 weeks of therapy, indicating a possible opportunity to interrogate a drug response over this time period. Further, having seen an effect with triple therapy, we were interested to explore the potential added value of showing an effect with the use of individual RTIs of the same class. In these ways, we hoped to maximize the amount of information that could be extracted from the small number of patients available for study, while minimizing the length of time—and burden for families—associated with participation in a clinical trial.

The crossover study design enabled participants to act as their own control and have the opportunity to take all three of the treatment regimes. Repeated measurements per patient maximized the amount of information available for analysis, which were fully utilized in the statistical analysis method. Thus, comparisons between each active treatment and ‘no treatment’ were mainly within‐patients rather than between‐patients. Nevertheless, we acknowledge that the interpretation of the results derived in the present study is limited by the low number of recruited patients, and by the difficulties experienced with drug compliance (especially in the ABC + 3TC + AZT arm).

There was insufficient evidence of the effectiveness of single therapy with ABC or 3TC in reducing type I IFN signalling over a 6‐week period in selected AGS genotypes, while not precluding the possibility that treatment over a longer period might be associated with an effect. In contrast to these non‐significant results, a statistically significant reduction of the IFN score was recorded after 3 weeks of triple therapy (where compliance was less of an issue than in the final 3 weeks of this drug arm). Even if this effect was not mirrored by a statistically significant reduction in IFN‐alpha protein levels, such a result is in keeping with the findings of our previous study.

The extreme value imputation method allowed us to test the sensitivity of the findings to the missing data, and this method has the advantage of covering all possible patterns of missing data (not just what we consider to be the most likely values). However, if results differ between the best‐case and worst‐case scenarios, this can make interpretation of the results challenging. Although the *p*‐value for the effect of triple therapy became non‐significant (*p* = 0.054) after applying this method, the mean difference and confidence intervals were similar to our primary analysis, suggesting that our results were fairly robust to our assumptions regarding the missing data.

The issue with compliance encountered in this trial, particularly relating to combined therapy comprising ABC + 3TC + AZT, is notable. Similar difficulties were experienced in our earlier trial, where 3 of 11 patients were unable to tolerate triple therapy over a 12‐month period. Indeed, this latter observation was one of the reasons prompting us to consider the use of single RTIs in a follow‐up study. More generally, the experience gained in the two studies suggests that the use of triple therapy in patients with AGS is challenging over the longer term.

Two patients, both mutated in *RNASEH2B* (both of whom were also being treated with the JAK1/2 inhibitor baricitinib), did not demonstrate an upregulation of type I IFN signalling at baseline, an observation in keeping with earlier studies.^4^ In our previous trial, such patients were excluded by prescreening, where a positive IFN score on at least three occasions in the 6 months before recruitment was stipulated as an inclusion criterion. For pragmatic reasons related to clinical trial regulations, a similar prescreening strategy was not feasible in this study. Of note, post hoc assessment of the primary outcome measure excluding these patients did not alter the results obtained (Table S5).

Even while noting the obvious impact of the COVID‐19 pandemic on non‐COVID‐19 clinical trials during 2020, the present study highlights structural issues, also noted by others^10^, ^11^ and particularly affecting paediatric studies,^12^ that severely affected trial prosecution. Thus, taking the dispatch of information packs to local sites in June 2021, the time to R&D approval at the four sites varied between 6 months and 21 months, with sponsor authorization taking a further 1 to 6 months thereafter. These non‐clinical delays contrast with the time to first patient screening after sponsor authorization to open, which was less than 3 months at all sites. Compounding the effect on patient enrolment at certain sites due to limited staffing capacity, authorization delays meant that recruitment at two sites was limited to a period of less than 6 months (i.e. 11% of an overall trial length of 54 months). Other non‐patient‐related problems encountered included the non‐availability of research MRI‐time for this study at one site, and a non‐negotiable decision of one associate medical director to deny the option to participants of MRI and lumbar puncture under sedation at another site. This latter decision resulted in a further 6‐month delay to site opening while permission was sought from the research ethics committee and MHRA for an amendment of the protocol that they had sanctioned in the prior 6 months. While the effect of these issues on the outcome of the present trial is not possible to determine, unless resolved, such structural difficulties clearly have implications for future rare disease experimental medicine approaches in the UK.^13^

## CONFLICT OF INTEREST STATEMENT

The authors declare the absence of any conflicts of interest, financial or otherwise.

## Supporting information


**Appendix S1:** Supplementary methods.

**Appendix S2:** Estimand for primary analysis.

**Figure S1:** Cartoon of study timeline.

**Figure S2:** Study timeline.

**Figure S3:** CONSORT flow diagram.

**Figure S4:** Summary treatment compliance data.

**Figure S5:** Correlation between paired interferon score and IFN‐alpha protein levels.

**Table S1:** Genotypes by participant.

**Table S2:** Complete data points by patient by visit.

**Table S3:** Summary statistics for IFN score at each time point split by randomized group.

**Table S4:** Sensitivity analysis of modelled assessment on primary outcome measure according to treatment arm with imputation of missing values.

**Table S5:** Post hoc analysis involving modelled assessment on primary outcome measure according to treatment arm excluding patients 11001 and 12002.

## Data Availability

Because of issues of patient confidentiality, no data are available beyond those in the published manuscript.
