# Supplementary material for: Reverse transcriptase inhibitors in Aicardi–Goutières syndrome: A crossover clinical trial
Source: Dev Med Child Neurol. 2024 Dec 4;67(6):750–7. doi: 10.1111/dmcn.16199 (PMC7617231; doi:10.1111/dmcn.16199)
Supplement: Supplementary file 1 — Appendix S1: Supplementary methods. [file DMCN-67-750-s009.docx]

**Appendix S1: SUPPLEMENTARY METHODS**

**INTERFERON SCORE**

For NanoString interferon stimulated gene (ISG) analysis, total RNA was extracted from whole blood with a PAXgene (PreAnalytix) RNA isolation kit. Analysis of 24 genes and 3 housekeeping genes was conducted using the NanoString custom CodeSet according to the manufacturer’s recommendations (NanoString Technologies). Agilent Tapestation was used to assess the quality of the RNA. Total RNA (200 ng) was loaded for each sample. Data were processed with nSolver software (NanoString Technologies). The data were normalized relative to the internal positive and negative calibrators, the three reference probes, and the control samples. The median of the 24 probes for each of 27 HC samples was calculated. The mean NanoString score of the 27 HCs +2 SD of the mean was calculated. Scores above this value (>2.724) were designated as positive. The list of probes used in the NanoString analysis is given below.

| **Probes of interest** (n = 24) | *IFI27*, *IFI44L*, *IFIT1*, *ISG15, RSAD2*, *SIGLEC1*, *CMPK2*, *DDX60*, *EPSTI1*, *FBXO39*, *HERC5*, *HES4*, *IFI44*, *IFI6*, *IFIH1*, *IRF7*, *LAMP3*, *LY6E*, *MX1*, *NRIR*, *OAS1*, *OASL*, *OTOF*, *SPATS2L* |
| --- | --- |
| **Reference probes** (n = 3) | *NRDC*, *OTUD5*, *TUBB* |

**SIMOA ASSAY FOR MEASUREMENT OF INTERFERON ALPHA PROTEIN**

Interferon alpha (IFNα) protein levels were quantified in plasma and CSF samples using a Simoa ultrasensitive digital ELISA homebrew assay (Quanterix, Billerica, MA, USA), in accordance with the manufacturer’s instructions, as previously described^1,2^.

The IFNα assay has specificity for all 14 IFNα subtypes^1^ and utilises two autoantibodies specific for IFN-α that were previously isolated and cloned from two APS1/APECED patients^3^. The 8H1 antibody clone functioned as the capture antibody after coating on paramagnetic beads (0.3 mg/ml). The 12H5 antibody clone was biotinylated (biotin/Ab ratio = 30:1) and used as the detector antibody. The SBG revelation enzyme concentration was 150 pM. Recombinant IFNα17/αI (Cat.No. 11150, PBL Assay Science, Piscataway, NJ, USA) was used to generate a standard curve. Plotting of the standard curve and interpolation of the unknown values from the standard curve were performed with GraphPad Prism (version 9.3.1.) using the Interpolate Values from Curves analysis and the Sigmoidal, 4PL, X is log(Concentration) equation. The limit of detection (LOD) was 0.16 fg/mL (background level + 2 SD). No sample had a signal below the LOD or above the saturation point of the assay.

For Simoa measurements, samples were randomly allocated to a plate position using a random number generator in Excel. All plasma and CSF samples for a specific participant were included within the same Simoa experiment to avoid batch effects. The plasma or CSF aliquots were thawed on wet ice, vortexed to ensure homogenisation and centrifuged at 14,000 g for 10 or 15 minutes at 4^o^C to remove debris. Supernatants were transferred to the wells of a 96-well plate, diluted 1/3 or 1/6 with Homebrew Detector/Sample Diluent (Quanterix) to avoid assay saturation, and incubated at room temperature for 1 hour before analysis. A two-person validation was performed to ensure that all samples were in the correct location according to the plate map. Each sample was measured in duplicate, following a 2-step ELISA configuration, on a Simoa HD-X Analyzer (Quanterix). A CV of <20% was considered acceptable. The results are expressed in fg/mL.

**References**

1. Rodero MP, Decalf J, Bondet V, et al. Detection of interferon alpha protein reveals differential levels and cellular sources in disease. J Exp Med 2017;214:1547–55.

2. Llibre A, Bondet V, Rodero M, et al. Development and Validation of an Ultrasensitive Single Molecule Array Digital Enzyme-linked Immunosorbent Assay for Human Interferon-α. J Vis Exp 2018;136:57421.

3. Meyer S, Woodward M, Hertel C, et al. AIRE-deficient patients harbor unique high-affinity disease-ameliorating autoantibodies. Cell 2016;166:582–95.

**CEREBRAL BLOOD FLOW**

Cerebral blood flow at rest was measured by non-contrast perfusion imaging with 3D pseudo continuous arterial spin labelling (ASL) MRI. Imaging analysis was undertaken using a GE advantage workstation, with the colour scale of cerebral blood flow maps set to rainbow. Regions of interest were drawn, selected visually on cerebral blood flow maps for each patient.
