# Supplementary material for: Reverse transcriptase inhibitors in Aicardi–Goutières syndrome: A crossover clinical trial
Source: Dev Med Child Neurol. 2024 Dec 4;67(6):750–7. doi: 10.1111/dmcn.16199 (PMC7617231; doi:10.1111/dmcn.16199)
Supplement: Supplementary file 2 — Appendix S2: Estimand for primary analysis. [file DMCN-67-750-s010.docx]

**Appendix S2: ESTIMAND for primary analysis**

Here we define the estimand for the analysis of the primary outcome analysis in the trial, in line with the addendum R1 to ICH E9 (Statistical Principles for Clinical Trials, <https://database.ich.org/sites/default/files/E9-R1_Step4_Guideline_2019_1203.pdf>, Defining the Appropriate Estimand for a Clinical Trial/Sensitivity Analyses).

**Population** Patients with Aicardi-Goutières syndrome (AGS) due to mutations in AGS1 = *TREX1*; AGS2 = *RNASEH2B*; AGS3 = *RNASEH2C*; AGS4 = *RNASEH2A*; AGS5 = *SAMHD1*; resident in the UK for more than 3 months and under 16 years of age. The primary analysis population will only include patients who received at least one of the active treatments (see section 4).

**Variable** IFN score

**Population-level Summary** Mean difference in IFN score between baseline (no treatment) and 6 weeks after treatment

The following **Intercurrent Events** have been identified which may change the interpretation of the measured primary outcome:

| **Event** | **Strategy** |
| --- | --- |
| 1. Discontinuation of IMP (investigational medicinal product) after it has been started when this is not due to intolerance or adverse event. | A pragmatic **treatment policy** **strategy** will be used to reflect the use of the active treatments in real clinical practice, where these treatments are taken. |
| 1. Discontinuation of IMP after it has been started due to intolerance or adverse event. | A pragmatic **treatment policy** **strategy** will be used to reflect the use of the active treatments in real clinical practice, where these treatments are taken. |
| 1. Concomitant illness unrelated to AGS condition. | A pragmatic **treatment policy** **strategy** will be used to reflect real clinical practice. |
| 1. Concomitant illness related to AGS condition. | A pragmatic **treatment policy** **strategy** will be used to reflect real clinical practice. |
| 1. Use of IMP in the no treatment arm. | A **hypothetical strategy** will be used, whereby patients who receive IMP in any of the no treatment periods will have their measurements changed to missing for all measurements taken during or after having received the incorrect IMP and before the next wash-out period (or end of follow-up). However, this event is expected to be extremely rare and not likely to occur. |
| 1. Use of incorrect IMP in a treatment arm (e.g. 3TC given to patient when it should have been ABC). | A **hypothetical strategy** will be used, whereby patients who receive an incorrect IMP in any of the treatment arms will have their follow-up measurements for the relevant treatment arm changed to missing (before the next wash-out period or end of follow-up). However, this event is expected to be extremely rare and is not likely to occur. |
| 1. Patient does not receive any IMP in any of the treatment arms. | A **principal stratum** **strategy** will be used, whereby patients who do not receive any IMP in any of the treatment arms are excluded from the analysis population. |
| 1. Patient does not receive any IMP in a particular treatment arm. | A **hypothetical strategy** will be used for patients who do NOT take *any* IMP in each of the treatment arms. In this case, they will have their measurements changed to missing for all measurements taken during the relevant treatment period and before the next wash-out period (or before end of follow-up). Note that if the patient takes at least some IMP in a particular treatment period (even if they discontinue), then a treatment policy approach will be used for that treatment arm as per events A and B. If the patient does not take any IMP in any of the treatment arms, then this is event G, and a principal stratum strategy will be used. |
| 1. Patient dies during the trial for whatever reason. | A **hypothetical** **strategy** will be used assuming the patient has not died. There will be no measurements of IFN score taken after the patient’s date of death. If the patient who died did not receive any of the active treatments, they will be excluded from the analysis population as per event G. No explicit imputation of missing values will be performed. |
| 1. Patient experiences a covid-19 infection. | A pragmatic **treatment policy** **strategy** will be used to reflect real clinical practice in the “post-covid world” where covid-19 infection can happen. In practice, very few patients are likely to contract covid-19 during the trial. |
| 1. Missed hospital visit. | This will usually lead to missing primary outcome values for a particular patient. A **hypothetical strategy** will be used assuming the patient did not miss their hospital visit. No explicit imputation of missing values will be performed. |
| 1. Hospital visit is not at the correct time. | All clinic visits should be within a window of up to seven days either side of the time designated for the clinic visit. If a clinic visit occurs outside of the visit window and data is collected at this visit, the data will still be used in the statistical analysis, taking into account the time at which data was collected. Therefore, we will use a **treatment policy** **strategy** for this intercurrent event. |
| 1. Patient takes additional medication. | A pragmatic **treatment policy** **strategy** will be used to reflect real clinical practice. |
| 1. Patient attends hospital or undergoes surgery for whatever reason. | A pragmatic **treatment policy** **strategy** will be used to reflect real life and real clinical practice. |

In summary, we will include all observed patient data in our analysis based on the target population, except that for which the patient did not take the IMP at all during a particular treatment arm where they should have taken IMP. Partial treatment adherence within a treatment arm will be considered as “treatment policy”. In a sensitivity analysis, we will consider a more hypothetical estimand where only observed data is included within a treatment arm if a patient was fully compliant to IMP throughout the relevant treatment period.
