# Supplementary figures and images for: Reverse transcriptase inhibitors in Aicardi–Goutières syndrome: A crossover clinical trial
Source: Dev Med Child Neurol. 2024 Dec 4;67(6):750–7. doi: 10.1111/dmcn.16199 (PMC7617231; doi:10.1111/dmcn.16199)

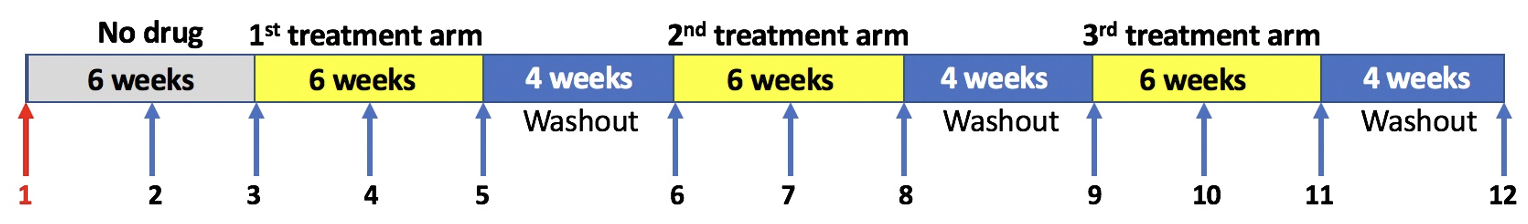

Supplement: Supplementary file 3 — Figure S1: Cartoon of study timeline. [file DMCN-67-750-s004.png]

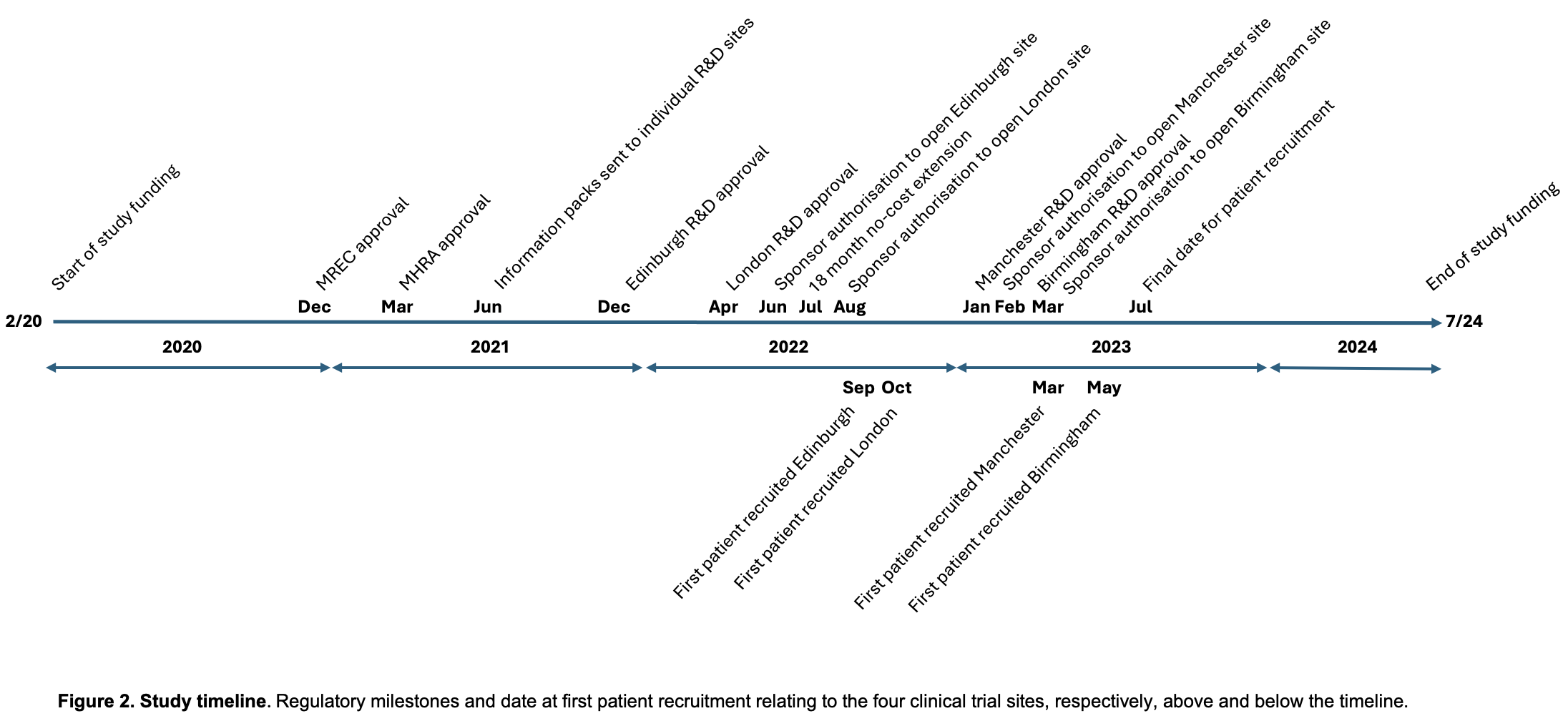

Supplement: Supplementary file 4 — Figure S2: Study timeline. [file DMCN-67-750-s001.png]

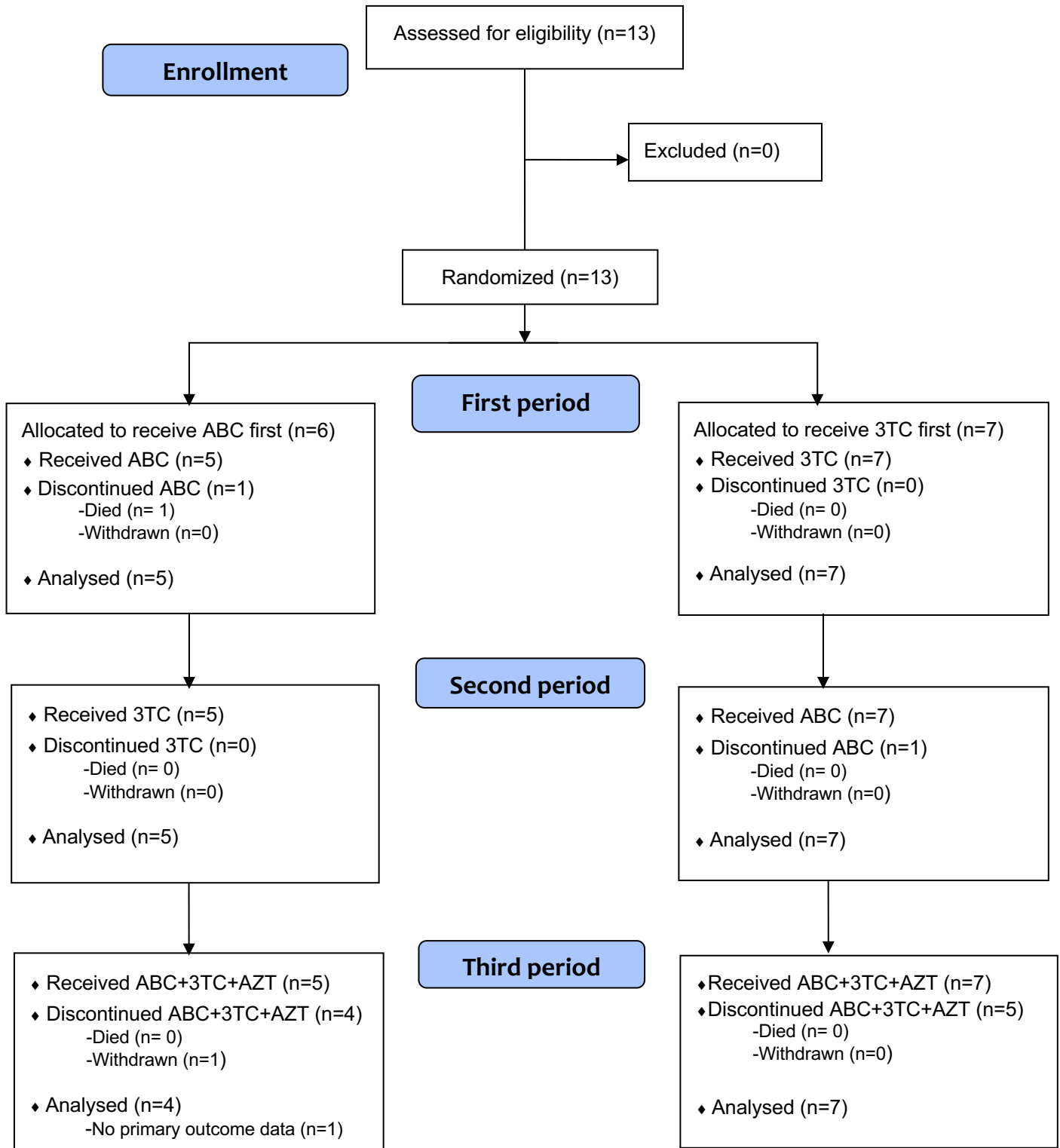

Supplement: Supplementary file 5 — Figure S3: CONSORT flow diagram. [file DMCN-67-750-s005.pdf]

**Figure S5. Correlation between paired interferon (IFN) score and IFN-alpha protein levels**


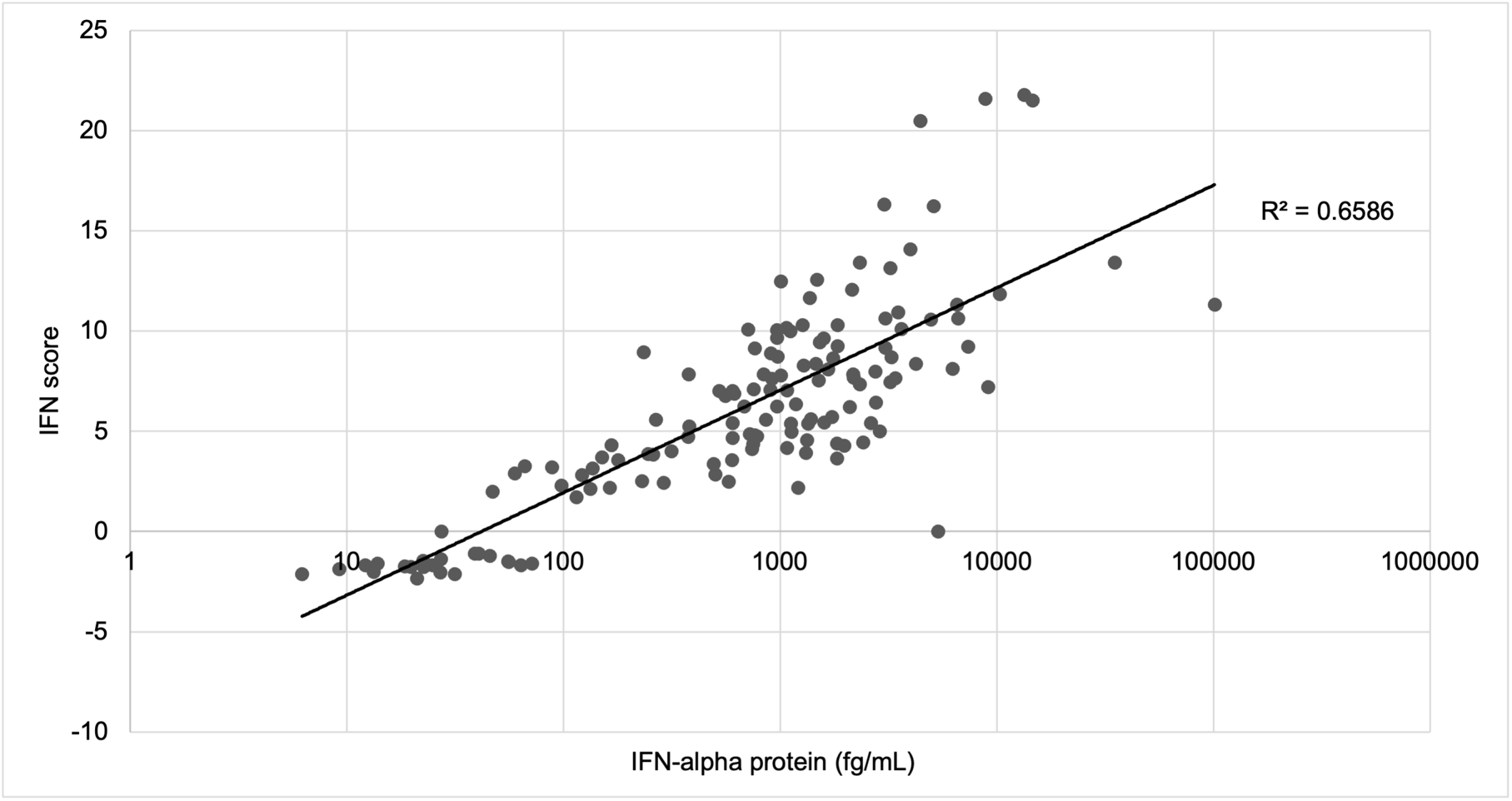

Supplement: Supplementary file 7 — Figure S5: Correlation between paired interferon score and IFN‐alpha protein levels. [file DMCN-67-750-s008.docx]
