# Supplementary material for: Reverse transcriptase inhibitors in Aicardi–Goutières syndrome: A crossover clinical trial
Source: Dev Med Child Neurol. 2024 Dec 4;67(6):750–7. doi: 10.1111/dmcn.16199 (PMC7617231; doi:10.1111/dmcn.16199)
Supplement: Supplementary file 6 — Figure S4: Summary treatment compliance data. [file DMCN-67-750-s006.docx]

**Figure S4**. **Summary treatment compliance data**. Treatment compliance assessed by treatment arm (ABC, 3TC, ABC+3TC+AZT) divided in weeks one to three (1-3) and weeks three to six (3-6). Green = good compliance (high treatment dose); ochre = reduced compliance (lower than expected treatment dose); red = poor compliance (extremely low, or no, dose of treatment received); black – patient died or full withdrawal from trial.

|  | **ABC (1-3)** | **ABC (3-6)** | **3TC (1-3)** | **3TC (3-6)** | **ABC+3TC+AZT (1-3)** | **ABC+3TC+AZT (3-6)** |
| --- | --- | --- | --- | --- | --- | --- |
| 1 |  |  |  |  |  |  |
| 2 |  |  |  |  |  |  |
| 3 |  |  |  |  |  |  |
| 4 |  |  |  |  |  |  |
| 5 |  |  |  |  |  |  |
| 6 |  |  |  |  |  |  |
| 7 |  |  |  |  |  |  |
| 8 |  |  |  |  |  |  |
| 9 |  |  |  |  |  |  |
| 10 |  |  |  |  |  |  |
| 11 |  |  |  |  |  |  |
| 12 |  |  |  |  |  |  |
| 13 |  |  |  |  |  |  |
