# Supplementary material for: Reverse transcriptase inhibitors in Aicardi–Goutières syndrome: A crossover clinical trial
Source: Dev Med Child Neurol. 2024 Dec 4;67(6):750–7. doi: 10.1111/dmcn.16199 (PMC7617231; doi:10.1111/dmcn.16199)
Supplement: Supplementary file 8 — Table S1: Genotypes by participant. [file DMCN-67-750-s002.docx]

**Table S1. Genotypes by participant**

| **Patient Number** | **Mutant genotype** | **Mutant allele 1** | **Mutant allele 2** |
| --- | --- | --- | --- |
| 1 | *SAMHD1* | c.452G>A, pGly151Glu | c.490C>T, p(Arg164Ter) |
| 2 | *TREX1* | c.341G>A / p.Arg114His | c.868_885del / p.Pro290_Ala295del |
| 3 | *RNASEH2B* | c.529G>A / p.(Ala177Thr) | c.509dup / p.Val17fs |
| 4 | *RNASEH2C* | c.205C>T / p.(Arg69Try) | c.205C>T / p.(Arg69Try) |
| 5 | *RNASEH2B* | c.529G>A / p.(Ala177Thr) | c.529G>A / p.(Ala177Thr) |
| 6 | *TREX1* | c.341G>A / p.Arg114His | c.341G>A / p.Arg114His |
| 7 | *SAMHD1* | c. 861T>A p.(Tyr287Ter) | c. 1343T>C p.(lle448Thr) |
| 8 | *RNASEH2C* | c.205C>T / p.(Arg69Try) | c.205C>T / p.(Arg69Try) |
| 9 | *RNASEH2B* | c.529G>A / p.(Ala177Thr) | c.132T>A p.Cys44* |
| 10 | *RNASEH2B* | c.529G>A / p.(Ala177Thr) | c.529G>A / p.(Ala177Thr) |
| 11 | *TREX1* | c.340C>T / p.Arg114Cys | c.340C>T / p.Arg114Cys |
| 12 | *SAMHD1* | c.625G>A / p.(Gly209Ser) | c.(1062_1063)_(1503_1504)del] |
| 13 | *RNASEH2B* | c.529G>A / p.(Ala177Thr) | c.31C>T / p.(Arg11Trp) |
