# Supplementary material for: Reverse transcriptase inhibitors in Aicardi–Goutières syndrome: A crossover clinical trial
Source: Dev Med Child Neurol. 2024 Dec 4;67(6):750–7. doi: 10.1111/dmcn.16199 (PMC7617231; doi:10.1111/dmcn.16199)
Supplement: Supplementary file 9 — Table S2: Complete data points by patient by visit. [file DMCN-67-750-s003.docx]

**Table S2. Complete data points by patient by visit.** Yellow = non-drug period, red = drug arm.

|  | **Patient number and visit** | **IFN-alpha protein** | **IFN score** | **Comment** | **CSF IFN-alpha protein** |
| --- | --- | --- | --- | --- | --- |
|  | 1v1 | **5346.993** | **Sample not obtained** |  |  |
| ***SAMHD1*** | 1v2 | **2168.882** | **7.85** |  |  |
|  | 1v3 | **2755.997** | **7.995** |  |  |
| **3TC (1-3 weeks)** | 1v4 | **3060.13** | **10.65** | **Good compliance** |  |
| **3TC (3-6 weeks)** | 1v5 | **7374.554** | **9.22** | **Good compliance** |  |
|  | 1v6 | **3634.757** | **10.12** |  |  |
| **ABC (1-3 weeks)** | 1v7 | **266.804** | **5.6** | **Good compliance** |  |
| **ABC (3-6 weeks)** | 1v8 | **246.103** | **3.89** | **Good compliance** |  |
|  | 1v9 | **765.27** | **4.825** |  |  |
| **ABC+3TC+AZT (1-3 weeks)** | 1v10 | **Sample not obtained** | **2.655** | **Good compliance** |  |
| **ABC+3TC+AZT (3-6 weeks)** | 1v11 | **374.889** | **4.745** | **Good compliance** |  |
|  | 1v12 | **2094.627** | **6.215** |  |  |
|  |  |  |  |  |  |
|  | 2v1 | **764.499** | **9.15** |  |  |
| ***TREX1*** | 2v2 | **1074.995** | **7.04** |  |  |
|  | 2v3 | **1268.466** | **10.31** |  |  |
| **3TC (1-3 weeks)** | 2v4 | **905.772** | **8.895** | **Good compliance** |  |
| **3TC (3-6 weeks)** | 2v5 | **1285.388** | **8.285** | **Good compliance** |  |
|  | 2v6 | **964.587** | **10.05** |  |  |
| **ABC (1-3 weeks)** | 2v7 | **860.029** | **5.59** | **Good compliance** |  |
| **ABC (3-6 weeks)** | 2v8 | **611.912** | **6.9** | **Good compliance** |  |
|  | 2v9 | **974.24** | **8.725** |  |  |
| **ABC+3TC+AZT (1-3 weeks)** | 2v10 | **1009.312** | **7.805** | **Reduced compliance** |  |
| **ABC+3TC+AZT (1-3 weeks)** | 2v11 | **1506.732** | **7.55** | **Reduced compliance** |  |
|  | 2v12 | **Sample not obtained** | **6.825** |  |  |
|  |  |  |  |  |  |
|  | 3v1 | **1750.344** | **8.64** |  |  |
| ***RNASEH2B*** | 3v2 | **2147.571** | **12.07** |  |  |
|  | 3v3 | **523.07** | **7.03** |  |  |
| **3TC (1-3 weeks)** | 3v4 | **4441.471** | **20.49** | **Good compliance** |  |
| **3TC (3-6 weeks)** | 3v5 | **492.111** | **3.375** | **Good compliance** |  |
|  | 3v6 | **967.8714521** | **6.25** |  |  |
| **ABC (1-3 weeks)** | 3v7 | **35031.507** | **13.43** | **Good compliance** |  |
| **ABC (3-6 weeks)** | 3v8 | **3977.262** | **14.1** | **Good compliance** |  |
|  | 3v9 | **5109.072** | **16.255** |  |  |
| **ABC+3TC+AZT (1-3 weeks)** | 3v10 | **379.236** | **5.245** | **Good compliance** |  |
| **ABC+3TC+AZT (3-6 weeks)** | 3v11 | **88.64** | **3.225** | **Good compliance** |  |
|  | 3v12 | **559.462** | **6.765** |  |  |
|  |  |  |  |  |  |
|  | 4v1 | **258.76** | **3.84** |  |  |
| ***RNASEH2C*** | 4v2 | **502.807** | **2.85** |  |  |
|  | 4v3 | **1385.542** | **5.625** |  |  |
| **ABC (1-3 weeks)** | 4v4 | **1462.869** | **8.375** | **Good compliance** |  |
| **ABC (3-6 weeks)** | 4v5 | **604.17** | **4.675** | **Good compliance** |  |
|  | 4v6 | **1824.73** | **4.395** |  |  |
| **3TC (1-3 weeks)** | 4v7 | **683.814** | **6.255** | **Good compliance** |  |
| **3TC (1-3 weeks)** | 4v8 | **1327.52** | **4.56** | **Good compliance** |  |
|  | 4v9 | **1122.052** | **5.4** |  |  |
|  | 4v10 | **150.751** | **3.71** |  |  |
| **ABC+3TC+AZT (1-3 weeks)** | 4v11 | **Sample not obtained** | **Sample not obtained** | Patient withdrawn from study |  |
| **ABC+3TC+AZT (3-6 weeks)** | 4v12 | **Sample not obtained** | **Sample not obtained** | Patient withdrawn from study |  |
|  |  |  |  |  |  |
|  | 5v1 | **378.329** | **7.85** |  |  |
| ***RNASEH2B*** | 5v2 | **1665.34** | **8.095** |  |  |
|  | 5v3 | **136.464** | **3.16** |  |  |
| **ABC (1-3 weeks)** | 5v4 | **Sample not obtained** | **Sample not obtained** | **No treatment taken** |  |
| **ABC (3-6 weeks)** | 5v5 | **Sample not obtained** | **Sample not obtained** | **No treatment taken** |  |
|  | 5v6 | **121.435** | **2.815** |  |  |
| **3TC (1-3 weeks)** | 5v7 | **178.632** | **3.57** | **Good compliance** |  |
| **3TC (3-6 weeks)** | 5v8 | **230.087** | **2.52** | **Good compliance** |  |
|  | 5v9 | **166.231** | **4.325** |  |  |
| **ABC+3TC+AZT (1-3 weeks)** | 5v10 | **2762.065** | **6.455** | **Reduced compliance** |  |
| **ABC+3TC+AZT (3-6 weeks)** | 5v11 | **Sample not obtained** | **Sample not obtained** | **No treatment taken** |  |
|  | 5v12 | **14575.9** | **21.51** |  |  |
|  |  |  |  |  |  |
|  | 6v1 | **2412.066** | **4.45** |  |  |
| ***TREX1*** | 6v2 | **1977.076** | **4.29** |  |  |
|  | 6v3 | **3403.123** | **7.66** |  |  |
| **3TC (1-3 weeks)** | 6v4 | **6239.444** | **8.13** | **Good compliance** |  |
| **3TC (3-6 weeks)** | 6v5 | **2617.089** | **5.425** | **Good compliance** |  |
|  | 6v6 | **1833.816646** | **3.645** |  |  |
| **ABC (1-3 weeks)** | 6v7 | **1738.079** | **5.74** | **Good compliance** |  |
| **ABC (3-6 weeks)** | 6v8 | **1317.076** | **3.93** | **Good compliance** |  |
|  | 6v9 | **9102.5** | **7.225** |  | **108047.8** |
| **ABC+3TC+AZT (1-3 weeks)** | 6v10 | **1209.4** | **2.18** | **Good compliance** |  |
| **ABC+3TC+AZT (3-6 weeks)** | 6v11 | **577.882** | **2.49** | **Good compliance** | **65776.49** |
|  | 6v12 | **2881.829** | **5** |  |  |
|  |  |  |  |  |  |
|  | 7v1 | **1069.42** | **10.175** |  |  |
| ***SAMHD1*** | 7v2 | **13371.05** | **21.785** |  |  |
|  | 7v3 | **1521.556** | **9.445** |  |  |
| **3TC (1-3 weeks)** | 7v4 | **1366.262** | **11.66** | **Good compliance** |  |
| **3TC (3-6 weeks)** | 7v5 | **709.337** | **10.1** | **Good compliance** |  |
|  | 7v6 | **2326.583** | **13.42** |  |  |
| **ABC (1-3 weeks)** | 7v7 | **1009.329** | **12.505** | **Good compliance** |  |
| **ABC (3-6 weeks)** | 7v8 | **1843.481** | **10.315** | **Good compliance** |  |
|  | 7v9 | **3019.713** | **16.32** |  |  |
| **ABC+3TC+AZT (1-3 weeks)** | 7v10 | **234.329** | **8.96** | **Poor compliance** |  |
| **ABC+3TC+AZT (3-6 weeks)** | 7v11 | **Sample not obtained** | **Sample not obtained** | **No treatment taken** |  |
|  | 7v12 | **8862.823** | **21.6** |  |  |
|  |  |  |  |  |  |
|  | 8v1 | **3229.465** | **7.46** |  |  |
| ***RNASEH2C*** | 8v2 | **1598.546** | **5.445** |  |  |
|  | 8v3 | **4219.126** | **8.365** |  |  |
| **ABC (1-3 weeks)** | 8v4 | **2179.333** | **7.7** | **Good compliance** |  |
| **ABC (3-6 weeks)** | 8v5 | **1179.266** | **6.355** | **Good compliance** |  |
|  | 8v6 | **3253.174** | **8.7** |  |  |
| Patient died | 8v7 | **Sample not obtained** | **Sample not obtained** | Patient died |  |
| Patient died | 8v8 | **Sample not obtained** | **Sample not obtained** | Patient died |  |
| Patient died | 8v9 | **Sample not obtained** | **Sample not obtained** | Patient died |  |
| Patient died | 8v10 | **Sample not obtained** | **Sample not obtained** | Patient died |  |
| Patient died | 8v11 | **Sample not obtained** | **Sample not obtained** | Patient died |  |
| Patient died | 8v12 | **Sample not obtained** | **Sample not obtained** | Patient died |  |
|  |  |  |  |  |  |
|  | 9v1 | **27.009** | **-2.02** | Baseline IFN score not elevated |  |
| ***RNASEH2B*** | 9v2 | **31.528** | **-2.1** | Baseline IFN score not elevated |  |
|  | 9v3 | **55.6** | **-1.485** | Baseline IFN score not elevated |  |
| **3TC (1-3 weeks)** | 9v4 | **71.434** | **-1.58** | Baseline IFN score not elevated |  |
| **3TC (3-6 weeks)** | 9v5 | **45.68** | **-1.19** | Baseline IFN score not elevated |  |
|  | 9v6 | **63.507** | **-1.675** | Baseline IFN score not elevated |  |
| **ABC (1-3 weeks)** | 9v7 | **55.85139414** | **-1.495** | Baseline IFN score not elevated |  |
| **ABC (3-6 weeks)** | 9v8 | **27.34** | **Sample not obtained** | Baseline IFN score not elevated |  |
|  | 9v9 | **21.09** | **-2.345** | Baseline IFN score not elevated |  |
| **ABC+3TC+AZT (1-3 weeks)** | 9v10 | **24.849** | **-1.665** | Baseline IFN score not elevated |  |
| **ABC+3TC+AZT (1-3 weeks)** | 9v11 | **22.66674165** | **-1.765** | Baseline IFN score not elevated |  |
|  | 9v12 | **9.21880367** | **-1.87** | Baseline IFN score not elevated |  |
|  |  |  |  |  |  |
|  | 10v1 | **163.53** | **2.18** |  |  |
| ***RNASEH2B*** | 10v2 | **114.741** | **1.735** |  |  |
|  | 10v3 | **97.691** | **2.305** |  | **6354.509** |
| **ABC (1-3 weeks)** | 10v4 | **741.67** | **4.115** | **Good compliance** |  |
| **ABC (3-6 weeks)** | 10v5 | **779.456** | **4.77** | **Good compliance** | **38987.13** |
|  | 10v6 | **599.101** | **3.585** |  |  |
| **3TC (1-3 weeks)** | 10v7 | **1074.018** | **4.185** | **Good compliance** |  |
| **3TC (3-6 weeks)** | 10v8 | **748.463** | **4.37** | **Good compliance** |  |
|  | 10v9 | **314.149** | **4.015** |  |  |
| **ABC+3TC+AZT (1-3 weeks)** | 10v10 | **132.57** | **2.13** | **Good compliance** |  |
| **ABC+3TC+AZT (3-6 weeks)** | 10v11 | **290.419** | **2.455** | **No treatment taken** |  |
|  | 10v12 | **46.902** | **2.01** |  |  |
|  |  |  |  |  |  |
|  | 11v1 | **6609.298** | **10.64** |  |  |
| ***TREX1*** | 11v2 | **3496.27** | **10.935** |  |  |
|  | 11v3 | **1843.407** | **9.25** |  |  |
| **ABC (1-3 weeks)** | 11v4 | **3228.587** | **13.155** | **Good compliance** |  |
| **ABC (3-6 weeks)** | 11v5 | **1478.949** | **12.585** | **Reduced compliance** |  |
|  | 11v6 | **1115.011** | **10** |  |  |
| **3TC (1-3 weeks)** | 11v7 | **4949.676** | **10.575** | **Good compliance** |  |
| **3TC (3-6 weeks)** | 11v8 | **3051.627** | **9.175** | **Good compliance** |  |
|  | 11v9 | **969.21** | **9.665** |  |  |
| **ABC+3TC+AZT (1-3 weeks)** | 11v10 | **6543.295** | **11.335** | **Good compliance** |  |
| **ABC+3TC+AZT (3-6 weeks)** | 11v11 | **101088.375** | **11.345** | **Reduced compliance** |  |
|  | 11v12 | **10329.78** | **11.86** |  |  |
|  |  |  |  |  |  |
|  | 12v1 | **603.95** | **7.02** |  |  |
| ***SAMHD1*** | 12v2 | **754.941** | **7.105** |  |  |
|  | 12v3 | **917.61** | **7.63** |  |  |
| **3TC (1-3 weeks)** | 12v4 | **1589.81** | **9.66** | **Good compliance** |  |
| **3TC (3-6 weeks)** | 12v5 | **Sample not obtained** | **Sample not obtained** | **Good compliance** |  |
|  | 12v6 | **902.301** | **7.09** |  |  |
| **ABC (1-3 weeks)** | 12v7 | **600.965** | **5.435** | **Good compliance** |  |
| **Reduced dose ABC (3-6 weeks)** | 12v8 | **1125.088** | **4.97** | **Reduced compliance** |  |
|  | 12v9 | **840.19** | **7.84** |  |  |
| **ABC+3TC+AZT (1-3 weeks)** | 12v10 | **724.629** | **4.87** | **Reduced compliance** |  |
| **ABC+3TC+AZT (3-6 weeks)** | 12v11 | **2323.032585** | **7.35** | **Reduced compliance** |  |
|  | 12v12 | **1345.581** | **5.39** |  |  |
|  |  |  |  |  |  |
|  | 13v1 | **13.25** | **-1.995** | Baseline IFN score not elevated |  |
| ***RNASEH2B*** | 13v2 | **12.128** | **-1.685** | Baseline IFN score not elevated |  |
|  | 13v3 | **27.216** | **-1.37** | Baseline IFN score not elevated |  |
| **ABC (1-3 weeks)** | 13v4 | **40.467** | **-1.095** | Baseline IFN score not elevated |  |
| **ABC (3-6 weeks)** | 13v5 | **59.66** | **2.925** | Baseline IFN score not elevated |  |
|  | 13v6 | **6.21** | **-2.11** | Baseline IFN score not elevated |  |
| **3TC (1-3 weeks)** | 13v7 | **66.405** | **3.28** | Baseline IFN score not elevated |  |
| **3TC (3-6 weeks)** | 13v8 | **38.925** | **-1.09** | Baseline IFN score not elevated |  |
|  | 13v9 | **18.539** | **-1.72** | Baseline IFN score not elevated |  |
| **ABC+3TC+AZT (1-3 weeks)** | 13v10 | **13.82** | **-1.575** | Baseline IFN score not elevated |  |
| **ABC+3TC+AZT (3-6 weeks)** | 13v11 | **22.5** | **-1.45** | Baseline IFN score not elevated |  |
|  | 13v12 | **19.756** | **-1.76** | Baseline IFN score not elevated |  |
