# Supplementary material for: Reverse transcriptase inhibitors in Aicardi–Goutières syndrome: A crossover clinical trial
Source: Dev Med Child Neurol. 2024 Dec 4;67(6):750–7. doi: 10.1111/dmcn.16199 (PMC7617231; doi:10.1111/dmcn.16199)
Supplement: Supplementary file 10 — Table S3: Summary statistics for IFN score at each time point split by randomized group. [file DMCN-67-750-s011.docx]

**Table S3. Summary statistics for IFN score at each time point split by randomised group**

|  | **ABC -> 3TC (N=6)** | **3TC -> ABC (N=7)** | **All** |
| --- | --- | --- | --- |
| **Timepoint 1 – Screening**  Mean (SD) | 5.0 (4.6) | 6.2 (4.5) | 5.6 (4.4) |
| Median [Q1-Q3] | 5.7 [2.2-7.9] | 7.8 [4.5-9.2] | 7.2 [3.0-8.9] |
| Min Max | -2.0,10.6 | -2.0,10.2 | -2.0,10.6 |
| N | 6 | 6 | 12 |
| **Timepoint 2 - No drug**  Mean (SD) | 4.6 (4.6) | 8.3 (7.3) | 6.6 (6.3) |
| Median [Q1-Q3] | 4.1 [1.7-8.1] | 7.1 [4.3-12.1] | 7.0 [2.9-8.1] |
| Min Max | -1.7,10.9 | -2.1,21.8 | -2.1,21.8 |
| N | 6 | 7 | 13 |
| **Timepoint 3 - No drug**  Mean (SD) | 4.6 (4.0) | 6.9 (3.9) | 5.8 (4.0) |
| Median [Q1-Q3] | 4.4 [2.3-8.4] | 7.7 [7.0-9.4] | 7.6 [3.2-8.4] |
| Min Max | -1.4,9.3 | -1.5,10.3 | -1.5,10.3 |
| N | 6 | 7 | 13 |
| **Timepoint 4 – Treatment 1: 3 weeks**  Mean (SD) | 6.5 (5.3) | 9.7 (6.5) | 8.3 (6.0) |
| Median [Q1-Q3] | 7.7 [4.1-8.4] | 9.7 [8.1-11.7] | 8.6 [5.9-11.2] |
| Min Max | -1.1,13.2 | -1.6,20.5 | -1.6,20.5 |
| N | 5 | 7 | 12 |
| **Timepoint 5 – Treatment 1: 6 weeks**  Mean (SD) | 6.3 (3.7) | 5.7 (4.5) | 6.0 (4.0) |
| Median [Q1-Q3] | 4.8 [4.7-6.4] | 6.9 [3.4-9.2] | 5.4 [3.4-9.2] |
| Min Max | 2.9,12.6 | -1.9,10.1 | -1.9,12.6 |
| N | 5 | 6 | 11 |
| **Timepoint 6 - Wash out**  Mean (SD) | 4.6 (4.4) | 7.0 (5.0) | 5.9 (4.7) |
| Median [Q1-Q3] | 4.0 [2.8-8.7] | 7.1 [3.6-10.1] | 6.3 [3.6-10.0] |
| Min Max | -2.1,10.0 | -1.7,13.4 | -2.1,13.4 |
| N | 6 | 7 | 13 |
| **Timepoint 7 – Treatment 2: 3 weeks**  Mean (SD) | 5.6 (3.0) | 6.7 (5.0) | 6.2 (4.2) |
| Median [Q1-Q3] | 4.2 [3.6-6.3] | 5.6 [5.4-12.5] | 5.6 [3.9-8.4] |
| Min Max | 3.3,10.6 | -1.5,13.4 | -1.5,13.4 |
| N | 5 | 7 | 12 |
| **Timepoint 8 – Treatment 2: 6 weeks**  Mean (SD) | 3.9 (3.7) | 7.4 (4.1) | 5.8 (4.1) |
| Median [Q1-Q3] | 4.4 [2.5-4.6] | 5.9 [3.9-10.3] | 4.6 [3.9-9.2] |
| Min Max | -1.1,9.2 | 3.9,14.1 | -1.1,14.1 |
| N | 5 | 6 | 11 |
| **Timepoint 9 - Wash out**  Mean (SD) | 4.3 (4.1) | 8.4 (6.5) | 6.7 (5.8) |
| Median [Q1-Q3] | 4.3 [4.0-5.4] | 7.8 [4.8-16.3] | 6.3 [4.2-9.2] |
| Min Max | -1.7,9.7 | -2.3,16.3 | -2.3,16.3 |
| N | 5 | 7 | 12 |
| **Timepoint 10 - ABC+3TC+AZT: 3 weeks**  Mean (SD) | 3.9 (5.4) | 4.3 (3.6) | 4.2 (4.1) |
| Median [Q1-Q3] | 2.9 [0.3-7.5] | 4.9 [2.2-7.8] | 3.7 [2.1-7.8] |
| Min Max | -1.6,11.3 | -1.7,9.0 | -1.7,11.3 |
| N | 4 | 7 | 11 |
| **Timepoint 11 - ABC+3TC+AZT: 6 weeks**  Mean (SD) | 4.7 (5.5) | 3.9 (3.5) | 4.2 (4.1) |
| Median [Q1-Q3] | 4.5 [0.5-8.9] | 4.0 [2.5-7.4] | 4.0 [2.5-7.4] |
| Min Max | -1.5,11.3 | -1.8,7.6 | -1.8,11.3 |
| N | 4 | 6 | 10 |
| **Timepoint 12 - Wash out**  Mean (SD) | 8.4 (10.5) | 7.1 (7.1) | 7.6 (7.9) |
| Median [Q1-Q3] | 6.9 [0.1-16.7] | 6.2 [5.0-6.8] | 6.2 [2.0-11.9] |
| Min Max | -1.8,21.5 | -1.9,21.6 | -1.9,21.6 |
| N | 4 | 7 | 11 |
