# Supplementary material for: Reverse transcriptase inhibitors in Aicardi–Goutières syndrome: A crossover clinical trial
Source: Dev Med Child Neurol. 2024 Dec 4;67(6):750–7. doi: 10.1111/dmcn.16199 (PMC7617231; doi:10.1111/dmcn.16199)
Supplement: Supplementary file 11 — Table S4: Sensitivity analysis of modelled assessment on primary outcome measure according to treatment arm with imputation of missing values. [file DMCN-67-750-s007.docx]

**Table S4. Sensitivity analysis of modelled assessment on primary outcome measure (interferon (IFN) score) according to treatment arm with imputation of missing values**

| **Comparison (vs no treatment)** | **Mean difference (active vs no treatment)** | **98.33% CI Lower** | **98.33% CI Upper** | **p-value** |
| --- | --- | --- | --- | --- |
| ABC at 3 weeks | 1.84 | -1.02 | 4.70 | 0.12 |
| ABC at 6 weeks | 0.62 | -2.80 | 2.92 | 0.96 |
| 3TC at 3 weeks | 0.33 | -2.55 | 3.19 | 0.79 |
| 3TC at 6 weeks | 1.72 | -1.17 | 4.61 | 0.15 |
| ABC+3TC+AZT at 3 weeks | -2.57 | -5.78 | 0.63 | 0.05 |
| ABC+3TC+AZT at 6 weeks | -1.13 | -4.41 | 2.15 | 0.40 |

ABC = abacavir; 3TC = lamivudine; AZT = zidovudine
