# Supplementary material for: Reverse transcriptase inhibitors in Aicardi–Goutières syndrome: A crossover clinical trial
Source: Dev Med Child Neurol. 2024 Dec 4;67(6):750–7. doi: 10.1111/dmcn.16199 (PMC7617231; doi:10.1111/dmcn.16199)
Supplement: Supplementary file 12 — Table S5: Post hoc analysis involving modelled assessment on primary outcome measure according to treatment arm excluding patients 11001 and 12002. [file DMCN-67-750-s012.docx]

**Table S5. Post-hoc analysis involving modelled assessment on primary outcome measure (interferon (IFN) score) according to treatment arm excluding patients 11001 and 12002 (mutated in *RNASEH2B* and not exhibiting an upregulation of type I IFN signalling at baseline)**

| **Comparison (vs no treatment)** | **Mean difference (active vs no treatment)** | **98.33% CI Lower** | **98.33% CI Upper** | **p-value** |
| --- | --- | --- | --- | --- |
| ABC at 3 weeks | 0.16 | -2.36 | 2.68 | 0.88 |
| ABC at 6 weeks | -0.8 | -3.39 | 1.68 | 0.42 |
| 3TC at 3 weeks | 1.55 | -0.96 | 4.06 | 0.14 |
| 3TC at 6 weeks | -1.72 | -4.36 | 0.92 | 0.12 |
| ABC+3TC+AZT at 3 weeks | -3.04 | -5.87 | -0.21 | 0.01 |
| ABC+3TC+AZT at 6 weeks | -2.41 | -5.44 | 0.62 | 0.06 |

ABC = abacavir; 3TC = lamivudine; AZT = zidovudine
