# Supplementary material for: Crossover trial of reverse transcriptase inhibitors in Aicardi-Goutières syndrome
Source: Dev Med Child Neurol. Author manuscript; Available in PMC 2025 Jun 1. (PMC7617231; doi:10.1111/dmcn.16199)
Supplement: Supplementary file 1 [file EMS201699-supplement-Supplementary.docx]

**Inhibition of reverse transcription in type I interferon mediated neuropathology** 

**AGS-RTI SUPPLEMENTARY FILE**

**CONTENTS**

|  | **Page** |
| --- | --- |
| **SECTION 1: ESTIMAND for the primary analysis** | **2** |
|  |  |
| **SECTION 2: SUPPLEMENTARY METHODS** | **5** |
|  |  |
| **SECTION 3: SUPPLEMENTARY FIGURES AND TABLES** | **7** |
| Figure S1. Summary compliance data | **7** |
| Figure S2. Correlation between paired interferon (IFN) score and IFN-alpha protein levels | **8** |
| Table S1. Genotypes by participant | **9** |
| Table S2. Complete data points by patient by visit | **10** |
| Table S3. Summary statistics for IFN score at each time point split by randomised group | **18** |
| Table S4. Sensitivity analysis of modelled assessment on primary outcome measure (interferon (IFN) score) according to treatment arm with imputation of missing values | **22** |
| Table S5. Post-hoc analysis involving modelled assessment on primary outcome measure (interferon (IFN) score) according to treatment arm excluding patients 11001 and 12002 (mutated in *RNASEH2B* and not exhibiting an upregulation of type I IFN signalling at baseline) | **23** |

**SECTION 1: ESTIMAND for primary analysis**

Here we define the estimand for the analysis of the primary outcome analysis in the trial, in line with the addendum R1 to ICH E9 (Statistical Principles for Clinical Trials, [https://database.ich.org/sites/default/files/E9-R1_Step4_Guideline_2019_1203.pdf](https://database.ich.org/sites/default/files/E9-R1_Step4_Guideline_2019_1203.pdf), Defining the Appropriate Estimand for a Clinical Trial/Sensitivity Analyses). 

**Population**	Patients with Aicardi-Goutières syndrome (AGS) due to mutations in AGS1 = *TREX1*; AGS2 = *RNASEH2B*; AGS3 = *RNASEH2C*; AGS4 = *RNASEH2A*; AGS5 = *SAMHD1*; resident in the UK for more than 3 months and under 16 years of age. The primary analysis population will only include patients who received at least one of the active treatments (see section 4). 

**Variable**	IFN score

**Population-level Summary**	Mean difference in IFN score between baseline (no treatment) and 6 weeks after treatment 

The following **Intercurrent Events** have been identified which may change the interpretation of the measured primary outcome:

| **Event** | **Strategy** |
| --- | --- |
| A. Discontinuation of IMP (investigational medicinal product) after it has been started when this is not due to intolerance or adverse event. | A pragmatic **treatment policy** **strategy** will be used to reflect the use of the active treatments in real clinical practice, where these treatments are taken. |
| B. Discontinuation of IMP after it has been started due to intolerance or adverse event. | A pragmatic **treatment policy** **strategy** will be used to reflect the use of the active treatments in real clinical practice, where these treatments are taken. |
| C. Concomitant illness unrelated to AGS condition. | A pragmatic **treatment policy** **strategy** will be used to reflect real clinical practice. |
| D. Concomitant illness related to AGS condition. | A pragmatic **treatment policy** **strategy** will be used to reflect real clinical practice. |
| E. Use of IMP in the no treatment arm. | A **hypothetical strategy** will be used, whereby patients who receive IMP in any of the no treatment periods will have their measurements changed to missing for all measurements taken during or after having received the incorrect IMP and before the next wash-out period (or end of follow-up). However, this event is expected to be extremely rare and not likely to occur. |
| F. Use of incorrect IMP in a treatment arm (e.g. 3TC given to patient when it should have been ABC). | A **hypothetical strategy** will be used, whereby patients who receive an incorrect IMP in any of the treatment arms will have their follow-up measurements for the relevant treatment arm changed to missing (before the next wash-out period or end of follow-up). However, this event is expected to be extremely rare and is not likely to occur. |
| G. Patient does not receive any IMP in any of the treatment arms. | A **principal stratum** **strategy** will be used, whereby patients who do not receive any IMP in any of the treatment arms are excluded from the analysis population. |
| H. Patient does not receive any IMP in a particular treatment arm. | A **hypothetical strategy** will be used for patients who do NOT take *any* IMP in each of the treatment arms. In this case, they will have their measurements changed to missing for all measurements taken during the relevant treatment period and before the next wash-out period (or before end of follow-up). Note that if the patient takes at least some IMP in a particular treatment period (even if they discontinue), then a treatment policy approach will be used for that treatment arm as per events A and B. If the patient does not take any IMP in any of the treatment arms, then this is event G, and a principal stratum strategy will be used. |
| I. Patient dies during the trial for whatever reason. | A **hypothetical** **strategy** will be used assuming the patient has not died. There will be no measurements of IFN score taken after the patient’s date of death. If the patient who died did not receive any of the active treatments, they will be excluded from the analysis population as per event G. No explicit imputation of missing values will be performed. |
| J. Patient experiences a covid-19 infection. | A pragmatic **treatment policy** **strategy** will be used to reflect real clinical practice in the “post-covid world” where covid-19 infection can happen. In practice, very few patients are likely to contract covid-19 during the trial. |
| K. Missed hospital visit. | This will usually lead to missing primary outcome values for a particular patient. A **hypothetical strategy** will be used assuming the patient did not miss their hospital visit. No explicit imputation of missing values will be performed. |
| L. Hospital visit is not at the correct time. | All clinic visits should be within a window of up to seven days either side of the time designated for the clinic visit. If a clinic visit occurs outside of the visit window and data is collected at this visit, the data will still be used in the statistical analysis, taking into account the time at which data was collected. Therefore, we will use a **treatment policy** **strategy** for this intercurrent event. |
| M. Patient takes additional medication. | A pragmatic **treatment policy** **strategy** will be used to reflect real clinical practice. |
| N. Patient attends hospital or undergoes surgery for whatever reason. | A pragmatic **treatment policy** **strategy** will be used to reflect real life and real clinical practice. |

In summary, we will include all observed patient data in our analysis based on the target population, except that for which the patient did not take the IMP at all during a particular treatment arm where they should have taken IMP. Partial treatment adherence within a treatment arm will be considered as “treatment policy”. In a sensitivity analysis, we will consider a more hypothetical estimand where only observed data is included within a treatment arm if a patient was fully compliant to IMP throughout the relevant treatment period.

**SECTION 2: SUPPLEMENTARY METHODS**

**INTERFERON SCORE**

For NanoString interferon stimulated gene (ISG) analysis, total RNA was extracted from whole blood with a PAXgene (PreAnalytix) RNA isolation kit. Analysis of 24 genes and 3 housekeeping genes was conducted using the NanoString custom CodeSet according to the manufacturer’s recommendations (NanoString Technologies). Agilent Tapestation was used to assess the quality of the RNA. Total RNA (200 ng) was loaded for each sample. Data were processed with nSolver software (NanoString Technologies). The data were normalized relative to the internal positive and negative calibrators, the three reference probes, and the control samples. The median of the 24 probes for each of 27 HC samples was calculated. The mean NanoString score of the 27 HCs +2 SD of the mean was calculated. Scores above this value (>2.724) were designated as positive. The list of probes used in the NanoString analysis is given below.

| **Probes of interest** (n = 24) | *IFI27*, *IFI44L*, *IFIT1*, *ISG15, RSAD2*, *SIGLEC1*, *CMPK2*, *DDX60*, *EPSTI1*, *FBXO39*, *HERC5*, *HES4*, *IFI44*, *IFI6*, *IFIH1*, *IRF7*, *LAMP3*, *LY6E*, *MX1*, *NRIR*, *OAS1*, *OASL*, *OTOF*, *SPATS2L* |
| --- | --- |
| **Reference probes** (n = 3) | *NRDC*, *OTUD5*, *TUBB* |

**SIMOA ASSAY FOR MEASUREMENT OF INTERFERON ALPHA PROTEIN**

Interferon alpha (IFNα) protein levels were quantified in plasma and CSF samples using a Simoa ultrasensitive digital ELISA homebrew assay (Quanterix, Billerica, MA, USA), in accordance with the manufacturer’s instructions, as previously described^1,2^. 

The IFNα assay has specificity for all 14 IFNα subtypes^1^ and utilises two autoantibodies specific for IFN-α that were previously isolated and cloned from two APS1/APECED patients^3^. The 8H1 antibody clone functioned as the capture antibody after coating on paramagnetic beads (0.3 mg/ml). The 12H5 antibody clone was biotinylated (biotin/Ab ratio = 30:1) and used as the detector antibody.  The SBG revelation enzyme concentration was 150 pM.  Recombinant IFNα17/αI (Cat.No. 11150, PBL Assay Science, Piscataway, NJ, USA) was used to generate a standard curve. Plotting of the standard curve and interpolation of the unknown values from the standard curve were performed with GraphPad Prism (version 9.3.1.) using the Interpolate Values from Curves analysis and the Sigmoidal, 4PL, X is log(Concentration) equation. The limit of detection (LOD) was 0.16 fg/mL (background level + 2 SD).  No sample had a signal below the LOD or above the saturation point of the assay.

For Simoa measurements, samples were randomly allocated to a plate position using a random number generator in Excel. All plasma and CSF samples for a specific participant were included within the same Simoa experiment to avoid batch effects. The plasma or CSF aliquots were thawed on wet ice, vortexed to ensure homogenisation and centrifuged at 14,000 g for 10 or 15 minutes at 4^o^C to remove debris.  Supernatants were transferred to the wells of a 96-well plate, diluted 1/3 or 1/6 with Homebrew Detector/Sample Diluent (Quanterix) to avoid assay saturation, and incubated at room temperature for 1 hour before analysis. A two-person validation was performed to ensure that all samples were in the correct location according to the plate map. Each sample was measured in duplicate, following a 2-step ELISA configuration, on a Simoa HD-X Analyzer (Quanterix). A CV of <20% was considered acceptable. The results are expressed in fg/mL. 

**References**

1. Rodero MP, Decalf J, Bondet V, et al. Detection of interferon alpha protein reveals differential levels and cellular sources in disease. J Exp Med 2017;214:1547–55.

2. Llibre A, Bondet V, Rodero M, et al. Development and Validation of an Ultrasensitive Single Molecule Array Digital Enzyme-linked Immunosorbent Assay for Human Interferon-α. J Vis Exp 2018;136:57421.

3. Meyer S, Woodward M, Hertel C, et al. AIRE-deficient patients harbor unique high-affinity disease-ameliorating autoantibodies. Cell 2016;166:582–95.

**CEREBRAL BLOOD FLOW**

Cerebral blood flow at rest was measured by non-contrast perfusion imaging with 3D pseudo continuous arterial spin labelling (ASL) MRI. Imaging analysis was undertaken using a GE advantage workstation, with the colour scale of cerebral blood flow maps set to rainbow. Regions of interest were drawn, selected visually on cerebral blood flow maps for each patient.

**SECTION 3: SUPPLEMENTARY FIGURES AND TABLES**

**Figure S1**. **Summary treatment compliance data**. Treatment compliance assessed by treatment arm (ABC, 3TC, ABC+3TC+AZT) divided in weeks one to three (1-3) and weeks three to six (3-6). Green = good compliance (high treatment dose); ochre = reduced compliance (lower than expected treatment dose); red = poor compliance (extremely low, or no, dose of treatment received); black – patient died or full withdrawal from trial. 

|  | **ABC (1-3)** | **ABC (3-6)** | **3TC (1-3)** | **3TC (3-6)** | **ABC+3TC+AZT (1-3)** | **ABC+3TC+AZT (3-6)** |
| --- | --- | --- | --- | --- | --- | --- |
| 1 |  |  |  |  |  |  |
| 2 |  |  |  |  |  |  |
| 3 |  |  |  |  |  |  |
| 4 |  |  |  |  |  |  |
| 5 |  |  |  |  |  |  |
| 6 |  |  |  |  |  |  |
| 7 |  |  |  |  |  |  |
| 8 |  |  |  |  |  |  |
| 9 |  |  |  |  |  |  |
| 10 |  |  |  |  |  |  |
| 11 |  |  |  |  |  |  |
| 12 |  |  |  |  |  |  |
| 13 |  |  |  |  |  |  |

**Figure S2. Correlation between paired interferon (IFN) score and IFN-alpha protein levels**

**Table S1. Genotypes by participant**

| **Patient Number** | **Mutant genotype** | **Mutant allele 1** | **Mutant allele 2** |
| --- | --- | --- | --- |
| 1 | *SAMHD1* | c.452G>A, pGly151Glu | c.490C>T, p(Arg164Ter) |
| 2 | *TREX1* | c.341G>A / p.Arg114His | c.868_885del / p.Pro290_Ala295del |
| 3 | *RNASEH2B* | c.529G>A / p.(Ala177Thr) | c.509dup / p.Val17fs |
| 4 | *RNASEH2C* | c.205C>T / p.(Arg69Try) | c.205C>T / p.(Arg69Try) |
| 5 | *RNASEH2B* | c.529G>A / p.(Ala177Thr) | c.529G>A / p.(Ala177Thr) |
| 6 | *TREX1* | c.341G>A / p.Arg114His | c.341G>A / p.Arg114His |
| 7 | *SAMHD1* | c. 861T>A p.(Tyr287Ter) | c. 1343T>C p.(lle448Thr) |
| 8 | *RNASEH2C* | c.205C>T / p.(Arg69Try) | c.205C>T / p.(Arg69Try) |
| 9 | *RNASEH2B* | c.529G>A / p.(Ala177Thr) | c.132T>A p.Cys44* |
| 10 | *RNASEH2B* | c.529G>A / p.(Ala177Thr) | c.529G>A / p.(Ala177Thr) |
| 11 | *TREX1* | c.340C>T / p.Arg114Cys | c.340C>T / p.Arg114Cys |
| 12 | *SAMHD1* | c.625G>A / p.(Gly209Ser) | c.(1062_1063)_(1503_1504)del] |
| 13 | *RNASEH2B* | c.529G>A / p.(Ala177Thr) | c.31C>T / p.(Arg11Trp) |

**Table S2. Complete data points by patient by visit.** Yellow = non-drug period, red = drug arm.

|  | **Patient number and visit** | **IFN-alpha protein** | **IFN score** | **Comment** | **CSF IFN-alpha protein** |
| --- | --- | --- | --- | --- | --- |
|  | 1v1 | **5346.993** | **Sample not obtained** |  |  |
| ***SAMHD1*** | 1v2 | **2168.882** | **7.85** |  |  |
|  | 1v3 | **2755.997** | **7.995** |  |  |
| **3TC (1-3 weeks)** | 1v4 | **3060.13** | **10.65** | **Good compliance** |  |
| **3TC (3-6 weeks)** | 1v5 | **7374.554** | **9.22** | **Good compliance** |  |
|  | 1v6 | **3634.757** | **10.12** |  |  |
| **ABC (1-3 weeks)** | 1v7 | **266.804** | **5.6** | **Good compliance** |  |
| **ABC (3-6 weeks)** | 1v8 | **246.103** | **3.89** | **Good compliance** |  |
|  | 1v9 | **765.27** | **4.825** |  |  |
| **ABC+3TC+AZT (1-3 weeks)** | 1v10 | **Sample not obtained** | **2.655** | **Good compliance** |  |
| **ABC+3TC+AZT (3-6 weeks)** | 1v11 | **374.889** | **4.745** | **Good compliance** |  |
|  | 1v12 | **2094.627** | **6.215** |  |  |
|  |  |  |  |  |  |
|  | 2v1 | **764.499** | **9.15** |  |  |
| ***TREX1*** | 2v2 | **1074.995** | **7.04** |  |  |
|  | 2v3 | **1268.466** | **10.31** |  |  |
| **3TC (1-3 weeks)** | 2v4 | **905.772** | **8.895** | **Good compliance** |  |
| **3TC (3-6 weeks)** | 2v5 | **1285.388** | **8.285** | **Good compliance** |  |
|  | 2v6 | **964.587** | **10.05** |  |  |
| **ABC (1-3 weeks)** | 2v7 | **860.029** | **5.59** | **Good compliance** |  |
| **ABC (3-6 weeks)** | 2v8 | **611.912** | **6.9** | **Good compliance** |  |
|  | 2v9 | **974.24** | **8.725** |  |  |
| **ABC+3TC+AZT (1-3 weeks)** | 2v10 | **1009.312** | **7.805** | **Reduced compliance** |  |
| **ABC+3TC+AZT (1-3 weeks)** | 2v11 | **1506.732** | **7.55** | **Reduced compliance** |  |
|  | 2v12 | **Sample not obtained** | **6.825** |  |  |
|  |  |  |  |  |  |
|  | 3v1 | **1750.344** | **8.64** |  |  |
| ***RNASEH2B*** | 3v2 | **2147.571** | **12.07** |  |  |
|  | 3v3 | **523.07** | **7.03** |  |  |
| **3TC (1-3 weeks)** | 3v4 | **4441.471** | **20.49** | **Good compliance** |  |
| **3TC (3-6 weeks)** | 3v5 | **492.111** | **3.375** | **Good compliance** |  |
|  | 3v6 | **967.8714521** | **6.25** |  |  |
| **ABC (1-3 weeks)** | 3v7 | **35031.507** | **13.43** | **Good compliance** |  |
| **ABC (3-6 weeks)** | 3v8 | **3977.262** | **14.1** | **Good compliance** |  |
|  | 3v9 | **5109.072** | **16.255** |  |  |
| **ABC+3TC+AZT (1-3 weeks)** | 3v10 | **379.236** | **5.245** | **Good compliance** |  |
| **ABC+3TC+AZT (3-6 weeks)** | 3v11 | **88.64** | **3.225** | **Good compliance** |  |
|  | 3v12 | **559.462** | **6.765** |  |  |
|  |  |  |  |  |  |
|  | 4v1 | **258.76** | **3.84** |  |  |
| ***RNASEH2C*** | 4v2 | **502.807** | **2.85** |  |  |
|  | 4v3 | **1385.542** | **5.625** |  |  |
| **ABC (1-3 weeks)** | 4v4 | **1462.869** | **8.375** | **Good compliance** |  |
| **ABC (3-6 weeks)** | 4v5 | **604.17** | **4.675** | **Good compliance** |  |
|  | 4v6 | **1824.73** | **4.395** |  |  |
| **3TC (1-3 weeks)** | 4v7 | **683.814** | **6.255** | **Good compliance** |  |
| **3TC (1-3 weeks)** | 4v8 | **1327.52** | **4.56** | **Good compliance** |  |
|  | 4v9 | **1122.052** | **5.4** |  |  |
|  | 4v10 | **150.751** | **3.71** |  |  |
| **ABC+3TC+AZT (1-3 weeks)** | 4v11 | **Sample not obtained** | **Sample not obtained** | Patient withdrawn from study |  |
| **ABC+3TC+AZT (3-6 weeks)** | 4v12 | **Sample not obtained** | **Sample not obtained** | Patient withdrawn from study |  |
|  |  |  |  |  |  |
|  | 5v1 | **378.329** | **7.85** |  |  |
| ***RNASEH2B*** | 5v2 | **1665.34** | **8.095** |  |  |
|  | 5v3 | **136.464** | **3.16** |  |  |
| **ABC (1-3 weeks)** | 5v4 | **Sample not obtained** | **Sample not obtained** | **No treatment taken** |  |
| **ABC (3-6 weeks)** | 5v5 | **Sample not obtained** | **Sample not obtained** | **No treatment taken** |  |
|  | 5v6 | **121.435** | **2.815** |  |  |
| **3TC (1-3 weeks)** | 5v7 | **178.632** | **3.57** | **Good compliance** |  |
| **3TC (3-6 weeks)** | 5v8 | **230.087** | **2.52** | **Good compliance** |  |
|  | 5v9 | **166.231** | **4.325** |  |  |
| **ABC+3TC+AZT (1-3 weeks)** | 5v10 | **2762.065** | **6.455** | **Reduced compliance** |  |
| **ABC+3TC+AZT (3-6 weeks)** | 5v11 | **Sample not obtained** | **Sample not obtained** | **No treatment taken** |  |
|  | 5v12 | **14575.9** | **21.51** |  |  |
|  |  |  |  |  |  |
|  | 6v1 | **2412.066** | **4.45** |  |  |
| ***TREX1*** | 6v2 | **1977.076** | **4.29** |  |  |
|  | 6v3 | **3403.123** | **7.66** |  |  |
| **3TC (1-3 weeks)** | 6v4 | **6239.444** | **8.13** | **Good compliance** |  |
| **3TC (3-6 weeks)** | 6v5 | **2617.089** | **5.425** | **Good compliance** |  |
|  | 6v6 | **1833.816646** | **3.645** |  |  |
| **ABC (1-3 weeks)** | 6v7 | **1738.079** | **5.74** | **Good compliance** |  |
| **ABC (3-6 weeks)** | 6v8 | **1317.076** | **3.93** | **Good compliance** |  |
|  | 6v9 | **9102.5** | **7.225** |  | **108047.8** |
| **ABC+3TC+AZT (1-3 weeks)** | 6v10 | **1209.4** | **2.18** | **Good compliance** |  |
| **ABC+3TC+AZT (3-6 weeks)** | 6v11 | **577.882** | **2.49** | **Good compliance** | **65776.49** |
|  | 6v12 | **2881.829** | **5** |  |  |
|  |  |  |  |  |  |
|  | 7v1 | **1069.42** | **10.175** |  |  |
| ***SAMHD1*** | 7v2 | **13371.05** | **21.785** |  |  |
|  | 7v3 | **1521.556** | **9.445** |  |  |
| **3TC (1-3 weeks)** | 7v4 | **1366.262** | **11.66** | **Good compliance** |  |
| **3TC (3-6 weeks)** | 7v5 | **709.337** | **10.1** | **Good compliance** |  |
|  | 7v6 | **2326.583** | **13.42** |  |  |
| **ABC (1-3 weeks)** | 7v7 | **1009.329** | **12.505** | **Good compliance** |  |
| **ABC (3-6 weeks)** | 7v8 | **1843.481** | **10.315** | **Good compliance** |  |
|  | 7v9 | **3019.713** | **16.32** |  |  |
| **ABC+3TC+AZT (1-3 weeks)** | 7v10 | **234.329** | **8.96** | **Poor compliance** |  |
| **ABC+3TC+AZT (3-6 weeks)** | 7v11 | **Sample not obtained** | **Sample not obtained** | **No treatment taken** |  |
|  | 7v12 | **8862.823** | **21.6** |  |  |
|  |  |  |  |  |  |
|  | 8v1 | **3229.465** | **7.46** |  |  |
| ***RNASEH2C*** | 8v2 | **1598.546** | **5.445** |  |  |
|  | 8v3 | **4219.126** | **8.365** |  |  |
| **ABC (1-3 weeks)** | 8v4 | **2179.333** | **7.7** | **Good compliance** |  |
| **ABC (3-6 weeks)** | 8v5 | **1179.266** | **6.355** | **Good compliance** |  |
|  | 8v6 | **3253.174** | **8.7** |  |  |
| Patient died | 8v7 | **Sample not obtained** | **Sample not obtained** | Patient died |  |
| Patient died | 8v8 | **Sample not obtained** | **Sample not obtained** | Patient died |  |
| Patient died | 8v9 | **Sample not obtained** | **Sample not obtained** | Patient died |  |
| Patient died | 8v10 | **Sample not obtained** | **Sample not obtained** | Patient died |  |
| Patient died | 8v11 | **Sample not obtained** | **Sample not obtained** | Patient died |  |
| Patient died | 8v12 | **Sample not obtained** | **Sample not obtained** | Patient died |  |
|  |  |  |  |  |  |
|  | 9v1 | **27.009** | **-2.02** | Baseline IFN score not elevated |  |
| ***RNASEH2B*** | 9v2 | **31.528** | **-2.1** | Baseline IFN score not elevated |  |
|  | 9v3 | **55.6** | **-1.485** | Baseline IFN score not elevated |  |
| **3TC (1-3 weeks)** | 9v4 | **71.434** | **-1.58** | Baseline IFN score not elevated |  |
| **3TC (3-6 weeks)** | 9v5 | **45.68** | **-1.19** | Baseline IFN score not elevated |  |
|  | 9v6 | **63.507** | **-1.675** | Baseline IFN score not elevated |  |
| **ABC (1-3 weeks)** | 9v7 | **55.85139414** | **-1.495** | Baseline IFN score not elevated |  |
| **ABC (3-6 weeks)** | 9v8 | **27.34** | **Sample not obtained** | Baseline IFN score not elevated |  |
|  | 9v9 | **21.09** | **-2.345** | Baseline IFN score not elevated |  |
| **ABC+3TC+AZT (1-3 weeks)** | 9v10 | **24.849** | **-1.665** | Baseline IFN score not elevated |  |
| **ABC+3TC+AZT (1-3 weeks)** | 9v11 | **22.66674165** | **-1.765** | Baseline IFN score not elevated |  |
|  | 9v12 | **9.21880367** | **-1.87** | Baseline IFN score not elevated |  |
|  |  |  |  |  |  |
|  | 10v1 | **163.53** | **2.18** |  |  |
| ***RNASEH2B*** | 10v2 | **114.741** | **1.735** |  |  |
|  | 10v3 | **97.691** | **2.305** |  | **6354.509** |
| **ABC (1-3 weeks)** | 10v4 | **741.67** | **4.115** | **Good compliance** |  |
| **ABC (3-6 weeks)** | 10v5 | **779.456** | **4.77** | **Good compliance** | **38987.13** |
|  | 10v6 | **599.101** | **3.585** |  |  |
| **3TC (1-3 weeks)** | 10v7 | **1074.018** | **4.185** | **Good compliance** |  |
| **3TC (3-6 weeks)** | 10v8 | **748.463** | **4.37** | **Good compliance** |  |
|  | 10v9 | **314.149** | **4.015** |  |  |
| **ABC+3TC+AZT (1-3 weeks)** | 10v10 | **132.57** | **2.13** | **Good compliance** |  |
| **ABC+3TC+AZT (3-6 weeks)** | 10v11 | **290.419** | **2.455** | **No treatment taken** |  |
|  | 10v12 | **46.902** | **2.01** |  |  |
|  |  |  |  |  |  |
|  | 11v1 | **6609.298** | **10.64** |  |  |
| ***TREX1*** | 11v2 | **3496.27** | **10.935** |  |  |
|  | 11v3 | **1843.407** | **9.25** |  |  |
| **ABC (1-3 weeks)** | 11v4 | **3228.587** | **13.155** | **Good compliance** |  |
| **ABC (3-6 weeks)** | 11v5 | **1478.949** | **12.585** | **Reduced compliance** |  |
|  | 11v6 | **1115.011** | **10** |  |  |
| **3TC (1-3 weeks)** | 11v7 | **4949.676** | **10.575** | **Good compliance** |  |
| **3TC (3-6 weeks)** | 11v8 | **3051.627** | **9.175** | **Good compliance** |  |
|  | 11v9 | **969.21** | **9.665** |  |  |
| **ABC+3TC+AZT (1-3 weeks)** | 11v10 | **6543.295** | **11.335** | **Good compliance** |  |
| **ABC+3TC+AZT (3-6 weeks)** | 11v11 | **101088.375** | **11.345** | **Reduced compliance** |  |
|  | 11v12 | **10329.78** | **11.86** |  |  |
|  |  |  |  |  |  |
|  | 12v1 | **603.95** | **7.02** |  |  |
| ***SAMHD1*** | 12v2 | **754.941** | **7.105** |  |  |
|  | 12v3 | **917.61** | **7.63** |  |  |
| **3TC (1-3 weeks)** | 12v4 | **1589.81** | **9.66** | **Good compliance** |  |
| **3TC (3-6 weeks)** | 12v5 | **Sample not obtained** | **Sample not obtained** | **Good compliance** |  |
|  | 12v6 | **902.301** | **7.09** |  |  |
| **ABC (1-3 weeks)** | 12v7 | **600.965** | **5.435** | **Good compliance** |  |
| **Reduced dose ABC (3-6 weeks)** | 12v8 | **1125.088** | **4.97** | **Reduced compliance** |  |
|  | 12v9 | **840.19** | **7.84** |  |  |
| **ABC+3TC+AZT (1-3 weeks)** | 12v10 | **724.629** | **4.87** | **Reduced compliance** |  |
| **ABC+3TC+AZT (3-6 weeks)** | 12v11 | **2323.032585** | **7.35** | **Reduced compliance** |  |
|  | 12v12 | **1345.581** | **5.39** |  |  |
|  |  |  |  |  |  |
|  | 13v1 | **13.25** | **-1.995** | Baseline IFN score not elevated |  |
| ***RNASEH2B*** | 13v2 | **12.128** | **-1.685** | Baseline IFN score not elevated |  |
|  | 13v3 | **27.216** | **-1.37** | Baseline IFN score not elevated |  |
| **ABC (1-3 weeks)** | 13v4 | **40.467** | **-1.095** | Baseline IFN score not elevated |  |
| **ABC (3-6 weeks)** | 13v5 | **59.66** | **2.925** | Baseline IFN score not elevated |  |
|  | 13v6 | **6.21** | **-2.11** | Baseline IFN score not elevated |  |
| **3TC (1-3 weeks)** | 13v7 | **66.405** | **3.28** | Baseline IFN score not elevated |  |
| **3TC (3-6 weeks)** | 13v8 | **38.925** | **-1.09** | Baseline IFN score not elevated |  |
|  | 13v9 | **18.539** | **-1.72** | Baseline IFN score not elevated |  |
| **ABC+3TC+AZT (1-3 weeks)** | 13v10 | **13.82** | **-1.575** | Baseline IFN score not elevated |  |
| **ABC+3TC+AZT (3-6 weeks)** | 13v11 | **22.5** | **-1.45** | Baseline IFN score not elevated |  |
|  | 13v12 | **19.756** | **-1.76** | Baseline IFN score not elevated |  |

**Table S3. Summary statistics for IFN score at each time point split by randomised group**

|  | **ABC -> 3TC (N=6)** | **3TC -> ABC (N=7)** | **All** |
| --- | --- | --- | --- |
| **Timepoint 1 – Screening** Mean (SD) | 5.0 (4.6) | 6.2 (4.5) | 5.6 (4.4) |
| Median [Q1-Q3] | 5.7 [2.2-7.9] | 7.8 [4.5-9.2] | 7.2 [3.0-8.9] |
| Min Max | -2.0,10.6 | -2.0,10.2 | -2.0,10.6 |
| N | 6 | 6 | 12 |
| **Timepoint 2 - No drug** Mean (SD) | 4.6 (4.6) | 8.3 (7.3) | 6.6 (6.3) |
| Median [Q1-Q3] | 4.1 [1.7-8.1] | 7.1 [4.3-12.1] | 7.0 [2.9-8.1] |
| Min Max | -1.7,10.9 | -2.1,21.8 | -2.1,21.8 |
| N | 6 | 7 | 13 |
| **Timepoint 3 - No drug** Mean (SD) | 4.6 (4.0) | 6.9 (3.9) | 5.8 (4.0) |
| Median [Q1-Q3] | 4.4 [2.3-8.4] | 7.7 [7.0-9.4] | 7.6 [3.2-8.4] |
| Min Max | -1.4,9.3 | -1.5,10.3 | -1.5,10.3 |
| N | 6 | 7 | 13 |
| **Timepoint 4 – Treatment 1: 3 weeks** Mean (SD) | 6.5 (5.3) | 9.7 (6.5) | 8.3 (6.0) |
| Median [Q1-Q3] | 7.7 [4.1-8.4] | 9.7 [8.1-11.7] | 8.6 [5.9-11.2] |
| Min Max | -1.1,13.2 | -1.6,20.5 | -1.6,20.5 |
| N | 5 | 7 | 12 |
| **Timepoint 5 – Treatment 1: 6 weeks** Mean (SD) | 6.3 (3.7) | 5.7 (4.5) | 6.0 (4.0) |
| Median [Q1-Q3] | 4.8 [4.7-6.4] | 6.9 [3.4-9.2] | 5.4 [3.4-9.2] |
| Min Max | 2.9,12.6 | -1.9,10.1 | -1.9,12.6 |
| N | 5 | 6 | 11 |
| **Timepoint 6 - Wash out** Mean (SD) | 4.6 (4.4) | 7.0 (5.0) | 5.9 (4.7) |
| Median [Q1-Q3] | 4.0 [2.8-8.7] | 7.1 [3.6-10.1] | 6.3 [3.6-10.0] |
| Min Max | -2.1,10.0 | -1.7,13.4 | -2.1,13.4 |
| N | 6 | 7 | 13 |
| **Timepoint 7 – Treatment 2: 3 weeks** Mean (SD) | 5.6 (3.0) | 6.7 (5.0) | 6.2 (4.2) |
| Median [Q1-Q3] | 4.2 [3.6-6.3] | 5.6 [5.4-12.5] | 5.6 [3.9-8.4] |
| Min Max | 3.3,10.6 | -1.5,13.4 | -1.5,13.4 |
| N | 5 | 7 | 12 |
| **Timepoint 8 – Treatment 2: 6 weeks** Mean (SD) | 3.9 (3.7) | 7.4 (4.1) | 5.8 (4.1) |
| Median [Q1-Q3] | 4.4 [2.5-4.6] | 5.9 [3.9-10.3] | 4.6 [3.9-9.2] |
| Min Max | -1.1,9.2 | 3.9,14.1 | -1.1,14.1 |
| N | 5 | 6 | 11 |
| **Timepoint 9 - Wash out** Mean (SD) | 4.3 (4.1) | 8.4 (6.5) | 6.7 (5.8) |
| Median [Q1-Q3] | 4.3 [4.0-5.4] | 7.8 [4.8-16.3] | 6.3 [4.2-9.2] |
| Min Max | -1.7,9.7 | -2.3,16.3 | -2.3,16.3 |
| N | 5 | 7 | 12 |
| **Timepoint 10 - ABC+3TC+AZT: 3 weeks** Mean (SD) | 3.9 (5.4) | 4.3 (3.6) | 4.2 (4.1) |
| Median [Q1-Q3] | 2.9 [0.3-7.5] | 4.9 [2.2-7.8] | 3.7 [2.1-7.8] |
| Min Max | -1.6,11.3 | -1.7,9.0 | -1.7,11.3 |
| N | 4 | 7 | 11 |
| **Timepoint 11 - ABC+3TC+AZT: 6 weeks** Mean (SD) | 4.7 (5.5) | 3.9 (3.5) | 4.2 (4.1) |
| Median [Q1-Q3] | 4.5 [0.5-8.9] | 4.0 [2.5-7.4] | 4.0 [2.5-7.4] |
| Min Max | -1.5,11.3 | -1.8,7.6 | -1.8,11.3 |
| N | 4 | 6 | 10 |
| **Timepoint 12 - Wash out** Mean (SD) | 8.4 (10.5) | 7.1 (7.1) | 7.6 (7.9) |
| Median [Q1-Q3] | 6.9 [0.1-16.7] | 6.2 [5.0-6.8] | 6.2 [2.0-11.9] |
| Min Max | -1.8,21.5 | -1.9,21.6 | -1.9,21.6 |
| N | 4 | 7 | 11 |

**Table S4. Sensitivity analysis of modelled assessment on primary outcome measure (interferon (IFN) score) according to treatment arm with imputation of missing values**

| **Comparison (vs no treatment)** | **Mean difference (active vs no treatment)** | **98.33% CI Lower** | **98.33% CI Upper** | **p-value** |
| --- | --- | --- | --- | --- |
| ABC at 3 weeks | 1.84 | -1.02 | 4.70 | 0.12 |
| ABC at 6 weeks | 0.62 | -2.80 | 2.92 | 0.96 |
| 3TC at 3 weeks | 0.33 | -2.55 | 3.19 | 0.79 |
| 3TC at 6 weeks | 1.72 | -1.17 | 4.61 | 0.15 |
| ABC+3TC+AZT at 3 weeks | -2.57 | -5.78 | 0.63 | 0.05 |
| ABC+3TC+AZT at 6 weeks | -1.13 | -4.41 | 2.15 | 0.40 |

ABC = abacavir; 3TC = lamivudine; AZT = zidovudine

**Table S5. Post-hoc analysis involving modelled assessment on primary outcome measure (interferon (IFN) score) according to treatment arm excluding patients 11001 and 12002 (mutated in** ***RNASEH2B*** **and not exhibiting an upregulation of type I IFN signalling at baseline)**

| **Comparison (vs no treatment)** | **Mean difference (active vs no treatment)** | **98.33% CI Lower** | **98.33% CI Upper** | **p-value** |
| --- | --- | --- | --- | --- |
| ABC at 3 weeks | 0.16 | -2.36 | 2.68 | 0.88 |
| ABC at 6 weeks | -0.8 | -3.39 | 1.68 | 0.42 |
| 3TC at 3 weeks | 1.55 | -0.96 | 4.06 | 0.14 |
| 3TC at 6 weeks | -1.72 | -4.36 | 0.92 | 0.12 |
| ABC+3TC+AZT at 3 weeks | -3.04 | -5.87 | -0.21 | 0.01 |
| ABC+3TC+AZT at 6 weeks | -2.41 | -5.44 | 0.62 | 0.06 |

ABC = abacavir; 3TC = lamivudine; AZT = zidovudine
